# Facioscapulohumeral muscular dystrophy: genetics, gene activation and downstream signalling with regard to recent therapeutic approaches: an update

**DOI:** 10.1186/s13023-021-01760-1

**Published:** 2021-03-12

**Authors:** Teresa Schätzl, Lars Kaiser, Hans-Peter Deigner

**Affiliations:** 1 Institute of Precision Medicine, Medical and Life Sciences Faculty, Furtwangen University, Jakob-Kienzle-Straße 17, 78054 Villingen-Schwenningen, Germany; 2 Institute of Pharmaceutical Sciences, University of Freiburg, Albertstraße 25, 79104 Freiburg i. Br., Germany; 3 EXIM Department, Fraunhofer Institute IZI, Leipzig, Schillingallee 68, 18057 Rostock, Germany; 4 Faculty of Science, Tuebingen University, Auf der Morgenstelle 8, 72076 Tübingen, Germany

**Keywords:** Facioscapulohumeral muscular dystrophy (FSHD), Double Homeobox 4 (*DUX4*), Epigenetic, Downstream signalling, Treatment strategies

## Abstract

Whilst a disease-modifying treatment for Facioscapulohumeral muscular dystrophy (FSHD) does not exist currently, recent advances in complex molecular pathophysiology studies of FSHD have led to possible therapeutic approaches for its targeted treatment. Although the underlying genetics of FSHD have been researched extensively, there remains an incomplete understanding of the pathophysiology of FSHD in relation to the molecules leading to *DUX4* gene activation and the downstream gene targets of *DUX4* that cause its toxic effects. In the context of the local proximity of chromosome 4q to the nuclear envelope, a contraction of the D4Z4 macrosatellite induces lower methylation levels, enabling the ectopic expression of *DUX4*. This disrupts numerous signalling pathways that mostly result in cell death, detrimentally affecting skeletal muscle in affected individuals. In this regard different options are currently explored either to suppress the transcription of *DUX4* gene, inhibiting *DUX4* protein from its toxic effects, or to alleviate the symptoms triggered by its numerous targets.

## Introduction

**Fig. 1:**
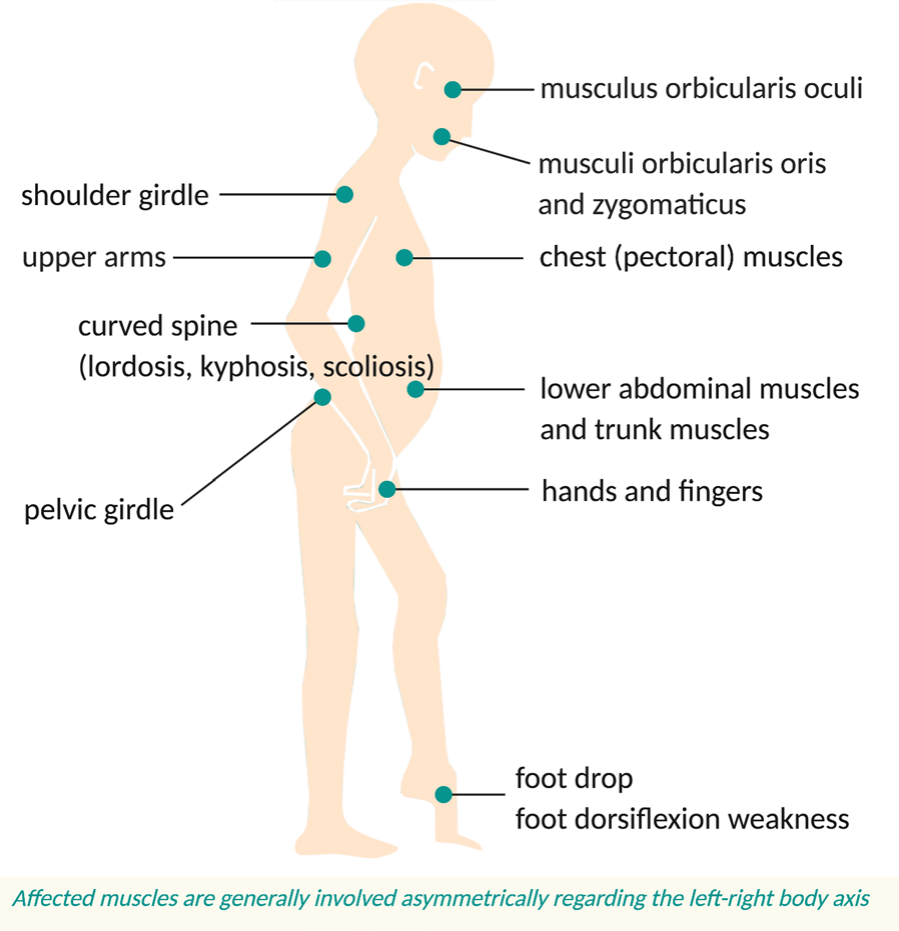
Overview of affected muscles in FSHD. The symptoms tend to start at the upper half of the body and then spread to lower body parts involving the pelvic girdle and the leg muscles. There is a high degree of clinical variability in disease severity and affected muscles are generally involved asymmetrically regarding the left–right body axis. In some cases, patients with foot drop can be supported by ankle–foot orthotics (AFOs) and knee-ankle–foot orthotics (KAFOs) [236]. Surgery to attach the scapula to the ribcage can enhance arm motion or alleviate pain [237–239]

Facioscapulohumeral muscular dystrophy (FSHD) is estimated to be the second most prevalent dystrophy after Duchenne muscular dystrophy [1] and affects approximately 870,000 people worldwide [2, 3]. However, the number of individuals with FSHD may be significantly higher because of undiagnosed cases [4]. FSHD is a genetic disease with symptoms that develop between infancy and late adulthood, and generally in the second decade of life [5]. Early onset can be seen as a marker for disease severity [6] and the disease is primarily characterized by asymmetric, progressive muscle weakness [7]. FSHD is inherited in an autosomal dominant pattern and the rate of de novo cases is estimated to be around 30%. There also appears to be a high frequency of somatic mosaicism [8]. Two types of FSHD have been reported, FSHD1 and FSHD2, which induce the same phenotype (see “Genetics”) [9]. In general, FSHD initially affects the upper half of the body, specifically the in “face (facio), shoulder girdle (scapulo), and upper arms (humeral)” [4]. As illustrated in Fig. 1, early symptoms are scapular winging (scapula alata) and inability to raise the arms above shoulder height. This is accompanied by problems in closing eyes or moving lips due to particularly affected musculi orbicularis oculi, oris and zygomaticus [10].

FSHD subsequently spreads to several other muscle areas. The disease can affect the distal leg muscles (by weak foot dorsiflexion), the abdominal wall and trunk muscles, and the pelvic muscles resulting in foot drop, difficulty when climbing stairs, and problems when straightening up from a horizontal position, respectively. Although FSHD is a slowly progressing muscle disorder [11], the associated physical limitations can be significant [9]. Moreover, respiratory issues—involving weak diaphragm and the expiratory abdominal muscles—are common in FSHD patients [12]. Respiratory function should therefore be evaluated during periodic clinical visits [13]. Nevertheless, most patients with FSHD have a normal life expectancy in contrast to those with Duchenne muscular dystrophy and Myotonic dystrophy [9]. FSHD generally begins at an approximate age of 20 years [14, 15]. The phenotype can range from minor restrictions to severe disability with disease severity varying widely between individuals [16]. This may explain the results of a cross-sectional study, by Hamel et al. (2019), involving 328 participants with FSHD which showed a high percentage of people experiencing a changed body image as disease burden (91.6%) besides factors such as physical limitations (96.9%) and pain (87.7%). Also, a substantial proportion (93.8%) of FSHD patients experience fatigue that significantly impacts their quality of life [17, 18]. Moreover, a qualitative study by Schipper et al. (2016), in which 25 FSHD patients suffering from severe fatigue (measured using the checklist individual strength (CIS) fatigue questionnaire) were interviewed, concluded that FSHD has a high influence on “participation, social contacts and quality of life” [19]. Chronic pain is another commonly described FSHD symptom [20], and is caused by overburdened joints and (asymmetric) muscle wasting that induces limb misalignments [21]. Nevertheless, a specific patient’s course of disease progression and muscle weakness is predictable because disease severity correlates with the number of D4Z4 repeats at chromosome 4 (see “Genetics”)*.* In fact, FSHD muscle weakness and progression is variable, even amongst siblings or between genders [4].

In case of early onset phenotype, which is estimated to occur in approximately 10% of disease carriers [22], patients usually show symptoms before the age of 5 [23] and are severely affected with rapid disease progression, marked muscular wasting, and weakness [24]. In this regard, comprehensive data on the clinical phenotype is missing. However, Goselink et al. (2017) conducted a systematic literature search on the clinical characteristics of early onset FSHD covering 43 articles with data on 227 patients. They found out 40% of patients were wheelchair-bound at the age of 18. Moreover, FSHD was frequently associated with extramuscular involvements, encompassing “hearing loss (40%), retinal abnormalities (37%) and developmental delay (8%)” showing a negative correlation between D4Z4 repeat size and disease severity, which is comparable with adult-onset FSHD (see “Genetics”)*.* Other research on the infantile phenotype showed symptoms such as cardiac arrhythmia, respiratory insufficiency, and difficulties with swallowing [25]. Therefore, a bilevel positive airway pressure machine or ventilator to initially manage symptomatic respiratory issues may be necessary [4].

### Genetics

There are two genetically distinct forms of FSHD known as FSHD1 and FSHD2. Although genetically distinct, both are the result of the inappropriate expression of a gene called *Double Homeobox 4* (*DUX4*) gene [26]. A 3.3 kilobase (kb) tandemly repeated sequence (D4Z4) located on chromosome 4q35 carries the gene [27]. It is usually expressed during embryogenesis, where it activates an early developmental program that marks the cleavage stage of embryogenesis [28–30] and is then effectively silenced [31]. While *DUX4* is expressed only in the testis [32] and in the thymus at low levels [33], it has detrimental effects when expressed in skeletal muscle resulting in FSHD [34].

Throughout the human genome there is a number of D4Z4-like sequences, mostly accompanying acrocentric chromosomes [35]. Also, a very homologous and equally polymorphic D4Z4 repeat is located on chromosome 10q26 (~ 98% similarity to the 4q35 locus), which has never been associated with FSHD [36] as this chromosome has no permissive single nucleotide polymorphism (SNP) in the *DUX4* Polyadenylation Signal (PAS) (see “DUX4 gene expression”) [26]*.* However, translocations between chromosomes 4 and 10 have been reported [37, 38], which complicates diagnosis (see “Methods of diagnosis”). Healthy individuals carry 11–100 D4Z4 repeats that are positioned within heterochromatin. Therefore, *DUX4* is not transcribed in somatic tissues [26]. FSHD1 patients show a reduced number of 1–10 repeats referred to as a "contraction" [9]. This contraction correlates with a “loss of repressive epigenetic marks” regarding methylation levels within the D4Z4 macrosatellite, enabling small molecules to trigger the ectopic expression of the *DUX4* gene in muscle cells [39]. The hypomethylation in FSHD is restricted to the D4Z4 repeat as it is not detected in the region proximal to the repeat [40].

Ninety-five percent of patients with FSHD have FSHD1, with the remaining percentage having FSHD2 [41]. In FSHD2 there is no contraction of the D4Z4 repeats [42] as the 4q35 locus contains 11–20 repeats. However, the number of repeats is not always decisive as some patients are excluded from this definition [43, 44]. In the case of FSHD2, a mutation in the *Structural Maintenance of Chromosomes flexible Hinge Domain Containing 1* (*SMCHD1*) gene (> 80% of FSHD2), or (rarely) in the De Novo* Methyltransferase 3B* (*DNMT3B*) gene leads to the hypomethylation of the D4Z4 array thereby enabling the aberrant expression of the *DUX4* protein [45, 46]. Recently, Hamanaka et al. (2020) also identified *LRIF1* as disease gene for FSHD2 [47] (see “FSHD2 and related diseases”).

**Fig. 2:**
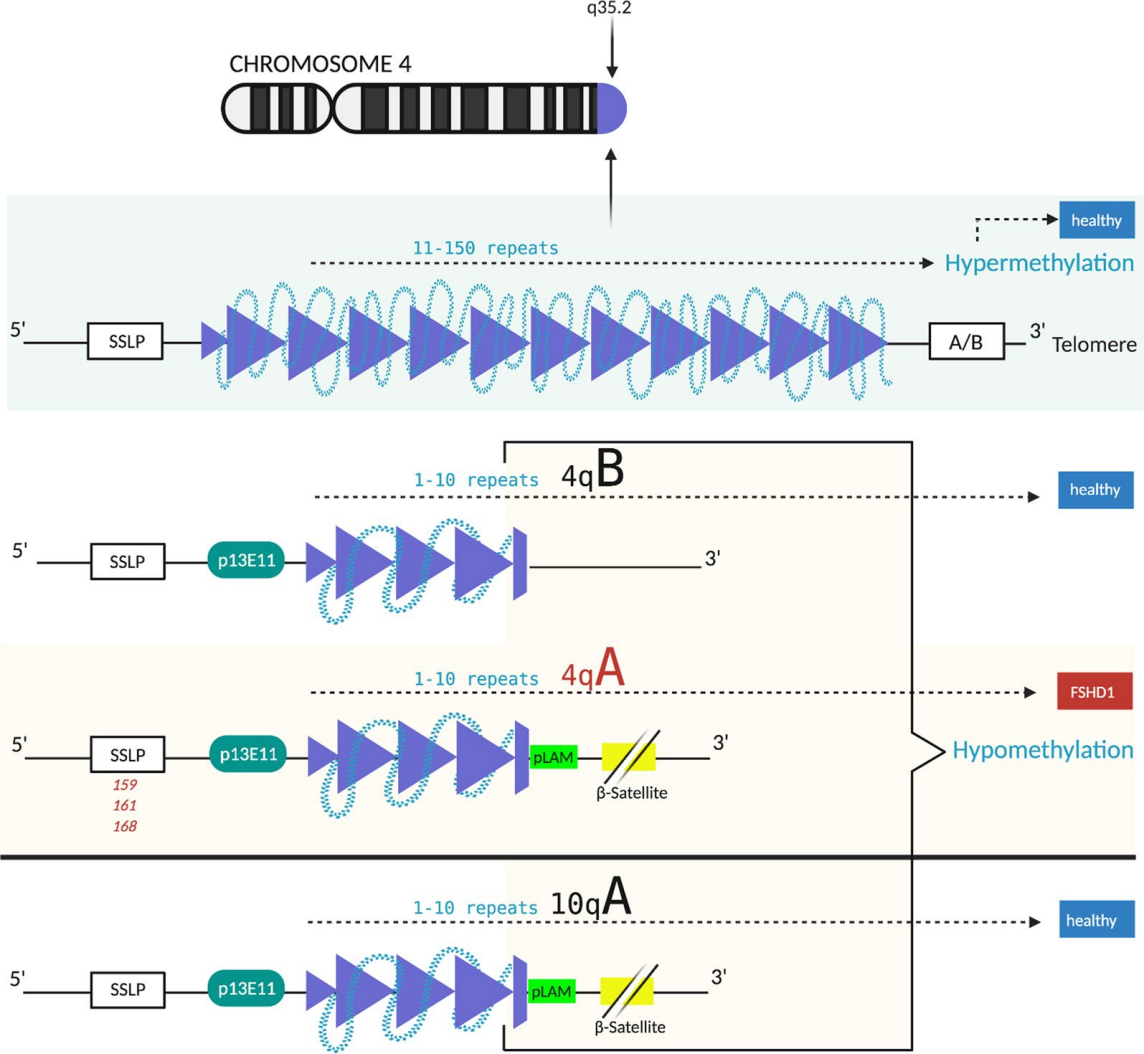
Chromosome 4q35.2 in healthy- and in FSHD1-individuals. 11–150 D4Z4 repeats (dark blue) leading to a high methylation level within the D4Z4 macrosatellite (light blue), which represses the transcription of *DUX4*. There are two possible haplotypes A and B, which are equally common and a Simple Sequence Length Polymorphism (SSLP) proximal to the D4Z4 repeat further classifying haplotype A or B [36]. FSHD only occurs in individuals, which carry the 4qA allele. People with contraction and thus minor methylation at chromosome 4q35 are either also carrying 4qA and have FSHD or 4qB and are healthy. The 4qA haplotype is further classified based on SSLPs proximal to the D4Z4 repeat. Only the common SSLP variant 4A161 and the rare variants 4A159 and 4A168 are reported to correlate with D4Z4 reduced alleles in FSHD patients. This example: 1–10 repeats/ FSHD1; the same conditions of a permissive Haplotype A apply for FSHD2 [36]. Haplotype A further carries the pLAM1 sequence (light green) and a beta-satellite repeat region immediately distal to the D4Z4 repeat (yellow). Detection is depicted via Southern Blot probe p13E-11 (dark green). Chromosome 10q26 is also illustrated as it shows nearly 100% similarity to the 4q35 locus [36]

A lower repeat number correlates with a more severe disease progression in patients with FSHD1 and 1–6 repeats. Most of the epigenetic factors that cause FSHD1 symptoms in patients with 7–10 repeats are unknown [48], and rare cases can be induced by an *SMCHD1* mutation although *SMCHD1* is commonly related to FSHD2 [49]. However, whilst there are the two major allelic forms 4qA and 4qB, only the former is associated with the disease. As illustrated in Fig. 2, the 4qA haplotype is further classified based on Simple Sequence Length Polymorphisms (SSLPs) proximal to the D4Z4 repeat. Only the SSLP variant 4A161 and the rare variants 4A159 and 4A168 were shown to correlate with D4Z4 reduced alleles in FSHD patients [36]. The 4qA sequence carries a 9 kb beta-satellite repeat region—“immediately distal to the D4Z4 repeat”—which cannot be found in 4qB [11]. The variant ATTAAA was discovered in the pLAM1 sequence of the 4qA alleles. This provides a PAS enabling the expression of the most distal copy of the *DUX4* gene [26, 50].

### Methylation levels of the D4Z4 macrosatellite in FSHD

FSHD methylation levels encompass both a low CpG methylation at D4Z4 DNA correlating with a reduced number of D4Z4 units [51–53] and a specific loss of H3K9me3 followed by the loss of *heterochromatin protein 1* (*HP1*) and cohesin binding at D4Z4. This suggests a more relaxed chromatin structure [54]. There seems to be a relationship between DNA hypomethylation and clinical severity in patients with an additional mutation in *SMCHD1* and *DNTM3B* [46, 55]. Furthermore, there are indications that infantile FSHD patients show extreme epigenetic dysregulation of the FSHD locus [56]. To date, different methylation analyses have been conducted [51–53, 57, 58]. However, the comparison of results proves difficult due to the evaluation of GpGs within different regions of the D4Z4 array and the use of different statistical tools. While Lemmers et al. (2015) consider the D4Z4 array as a linear string of mathematical units to define global methylation [48, 52], Calandra et al. (2016) highlight the potential of one single CPG to distinguish individuals and point to the CpGs distal to the D4Z4 array [57]. Other study findings doubt that D4Z4 methylation mirrors the clinical expression of FSHD [59] and indicate that measurement of this epigenetic mark must be interpreted with caution in clinical practice [58]. According to the hypothesis of Gaillard et al. (2019), FSHD chromatin landscape is not inherited but progressively installed upon differentiation. Based on confocal imaging the authors showed long-distance interactions between the D4Z4 array, the telomere and the nuclear lamina (see “FAT1 in the context of long distance interactions”) [60]. The nuclear lamina impact on global chromatin architecture is poorly understood. Experiments in mice and Drosophila have already shown its major role in chromatin organisation shaping the 3D genome [61, 62]. In this context, as chromatin regulation involves long-distance interactions [63], the entire location of the chromosome 4 within the nucleus should be looked at more closely.

### FSHD2 and related diseases

FSHD types 1 and 2 show a common pathomechanism that results from the stabilization of the *DUX4* transcript. Prerequisites for FSHD2 are a mutant *SMCHD1-* or (rarely) *DNMT3B* allele and a permissive 4qA allele [45, 46]. In this case, the D4Z4 repeats on both 4q35 copies and on chromosome 10 are hypomethylated [64]. Moreover, Hamanaka et al. (2020) recently identified *LRIF1* as a rare FSHD2 disease gene, which was also shown to bind to the D4Z4 repeat array. Knockdown of the *LRIF1* long isoform resulted in *DUX4* expression as a result of partial chromatin relaxation. Interestingly, while almost all patients with FSHD2 show monoallelic mutations in *SMCHD1* or *DNMT3B*, *LRIF1* mutation was demonstrated to be biallelic. According to the authors, *LRIF1* mutations are a rare reason for FSHD and should thus only be considered in FSHD2 when tested negative for *SMCHD1* mutations [47].

Interestingly, D4Z4 hypomethylation is not specific to FSHD. In this regard, mutations of the two FSHD2 modifiers—*SMCHD1* and *DNMT3B*—could be reported in other diseases as well. Missense mutations in *SMCHD1* were shown to cause *Bosma arhinia microphthalmia syndrome* (BAMS) [65, 66], which is a very rare disease characterized by complete absence of the nose and possible ocular defects [66]. In contrast to FSHD2 individuals, BAMS patients show no signs of muscular dystrophy while *SMCHD1* mutations in FSHD2 are not associated with craniofacial defects, which are characteristic for BAMS. Both diseases induce identical transcription of the *DUX4* gene and increased expression of some of its target genes. However, there are different mutations in *SMCHD1,* which seem to have no impact on transcription, but on the epigenetic organisation of D4Z4, thus showing entirely different phenotypical outcomes [59]. *SMCHD1* is involved in repairing DNA double-strand breaks [67] and in the epigenetic regulation of different genes particularly mediated by the histone mark H3K9me3 [68]. Furthermore, it plays a role in inactivating the X chromosome. In case of FSHD2 or BAMS, mutations do not affect X inactivation. While in most BAMS patients mutations induce gain of ATPase activity, there is a loss of function in FSHD2 regarding remethylation [59] (see “The ATPase domain of BAMS and FSHD2”). Intriguingly, the latter was found to be similar to what is observed in cells from patients with immunodeficiency, centromeric instability, and facial anomalies (ICF) syndrome [59, 69] as one of its subtypes, ICF1, arises from *DNMT3B* mutations. ICF1 is associated with reduced levels or absence of serum immunoglobulins [70] and it affects facial appearance [46]. In contrast to FSHD2, which is caused by dominant mutation of *DNMT3B*, ICF1 is inherited in an autosomal recessive pattern. Whereas heterozygous ICF1-mutation carriers neither show any muscle dystrophy nor present immunological abnormalities, in FSHD2 the absence of an immunological phenotype could be described by the presence of one wild-type *DNMT3B* allele. A low D4Z4-repeat size and a permissive 4qA allele containing a *DUX4* PAS were reported to facilitate *DUX4* expression when both *DNMT3B* alleles are mutated. However, in this case, next to the described features of ICF1, muscle weakness has never been reported. According to van den Boogaard et al. (2016), the short life expectancy of ICF1 individuals might be the reason for the lacking FSHD2 phenotype [46].

#### The ATPase domain of BAMS and FSHD2

In FSHD2, specific *SMCHD1* mutations have been described across the whole coding sequence [59], whereas mutations inducing BAMS are localized within exons 3 to 13 [66]. In this context, the ATPase domain of *SMCHD1* is an overlapping area of mutations in both diseases. However, whilst FSHD2-specific variants are typically localised in the ATP binding pocket, BAMS variants are most often positioned at the dimer interface—being an area that may be essential for the dimerisation of the ATPase domain [71]. However, overlapping variants have also been discovered amongst the different mutations. Nevertheless, the individual diseases seem to be mutually exclusive as studies of extended FSHD2 families carrying BAMS-specific variants have shown no signs or symptoms characteristic for BAMS. Interestingly, one BAMS patient with FSHD symptoms, having a moderately sized D4Z4 repeat on a 4qA allele, was found [11, 65]. Lemmers et al. (2019) suggested that BAMS might also incorporate another yet unknown locus [71]. But the mechanisms are still not fully understood and require further structural and biochemical analysis.

### *DUX4* protein

Tassin et al. (2013) presented a model of how an initially very low concentration of the *DUX4* protein can potentiate its effects. After the *DUX4* gene is activated, it is transcribed into mRNA, which is translocated into the cytoplasm domain near the activated nucleus. Then it is translated into the *DUX4* protein, which carries a nuclear localization signal (NLS). It diffuses in the cytoplasm and is transported into various neighbouring nuclei. Subsequently, the cascade initiation and amplification begins, as *DUX4* activates other transcription factors which are imported into neighbouring nuclei. The number of activated nuclei and expressed genes grows at every point, thereby enabling an amplification of the initial trigger [72].

**Fig. 3:**
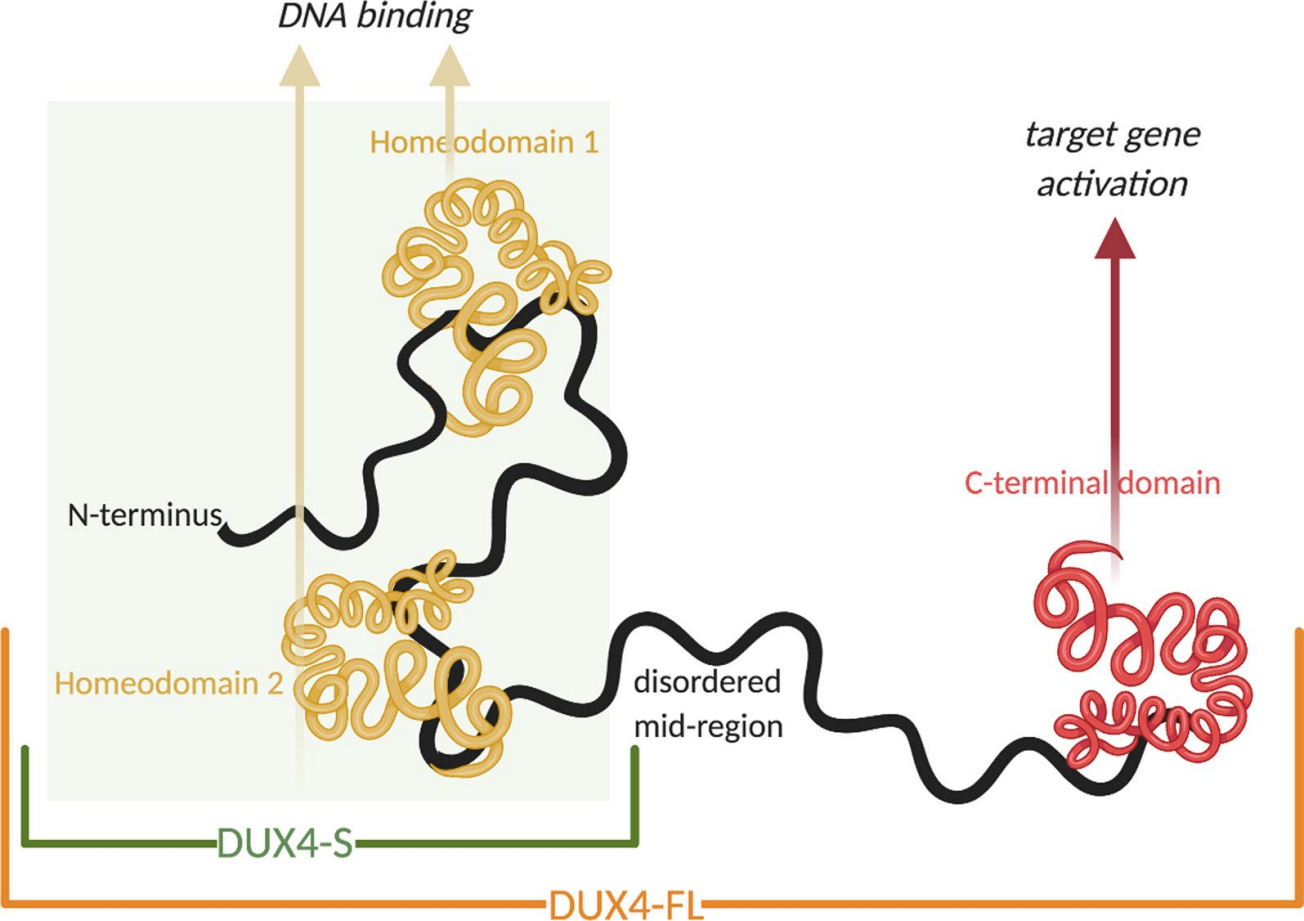
*DUX4*-FL protein. This protein contains 424 amino acids and is expected to have well-defined tertiary structures in each of the two DNA-binding homeodomains (amino acids 19–79 and 94–154) and in the most C-terminal region (amino acids ∼ 365–424). The C-terminal region includes the transcription-activating domain (TAD) and a *p300*-binding domain. The region between the second homeodomain and the C-terminal domain (amino acids ∼ 155–364) is predicted to be disordered. The protein contains a potential nine amino acid TAD (9aaTAD) at amino acids 371–379 (classified as a 92% match) [74]

Snider et al. (2010) showed in vitro that FSHD muscle expresses another splice form of *DUX4* mRNA when compared with control muscle. Figure 3 shows that control muscle generates low amounts of a splice form of *DUX4* encoding the amino-terminal part and both homeodomains, but it does not contain the C-terminal domain. This is referred to as *DUX4-S* (S for *short*) and is not toxic [73]. In contrast, FSHD muscle produces *DUX4-FL* (FL for *full-lengths*) mRNA that encodes the whole *DUX4* protein, which contains 424 amino acids [32]. Previous in vitro studies showed that *DUX4-*induced pathology requires both intact homeodomains and a transcription-activating domain (TAD) in the C-terminal region of the protein. Furthermore, it was found that non-toxic constructs with both homeodomains intact could act as inhibitors of *DUX4* transcriptional activation, and is likely due to competition for promoter sites [74].

The two homeoboxes situated at the N-terminus of the protein are responsible for binding to the DNA. The C-terminal domain is relevant for target gene activation and contributes to the cellular toxicity of *DUX4-FL* by interacting with histone acetyltransferase *p300* and transcriptional coactivator *CBP* [75, 76] (see “DUX4 Downstream Signalling: p300”).

Zhang et al. 2016 analysed the DNA-binding sequence specificity of *DUX4* by using Chromatin ImmunoPrecipitation DNA-Sequencing (ChIP-seq) analysis and identified a consensus containing two tandem TAAT motifs (TAAT[T/C][T/C]AATCA). They showed that all four variants could be identified by *DUX4*, but the motif containing a central cytosine followed by a thymidine (TAATCTAATCA) was the most preferred by *DUX4*, thus having the greatest transcriptional activity in vivo [77].

### *DUX4* gene expression

**Fig. 4:**
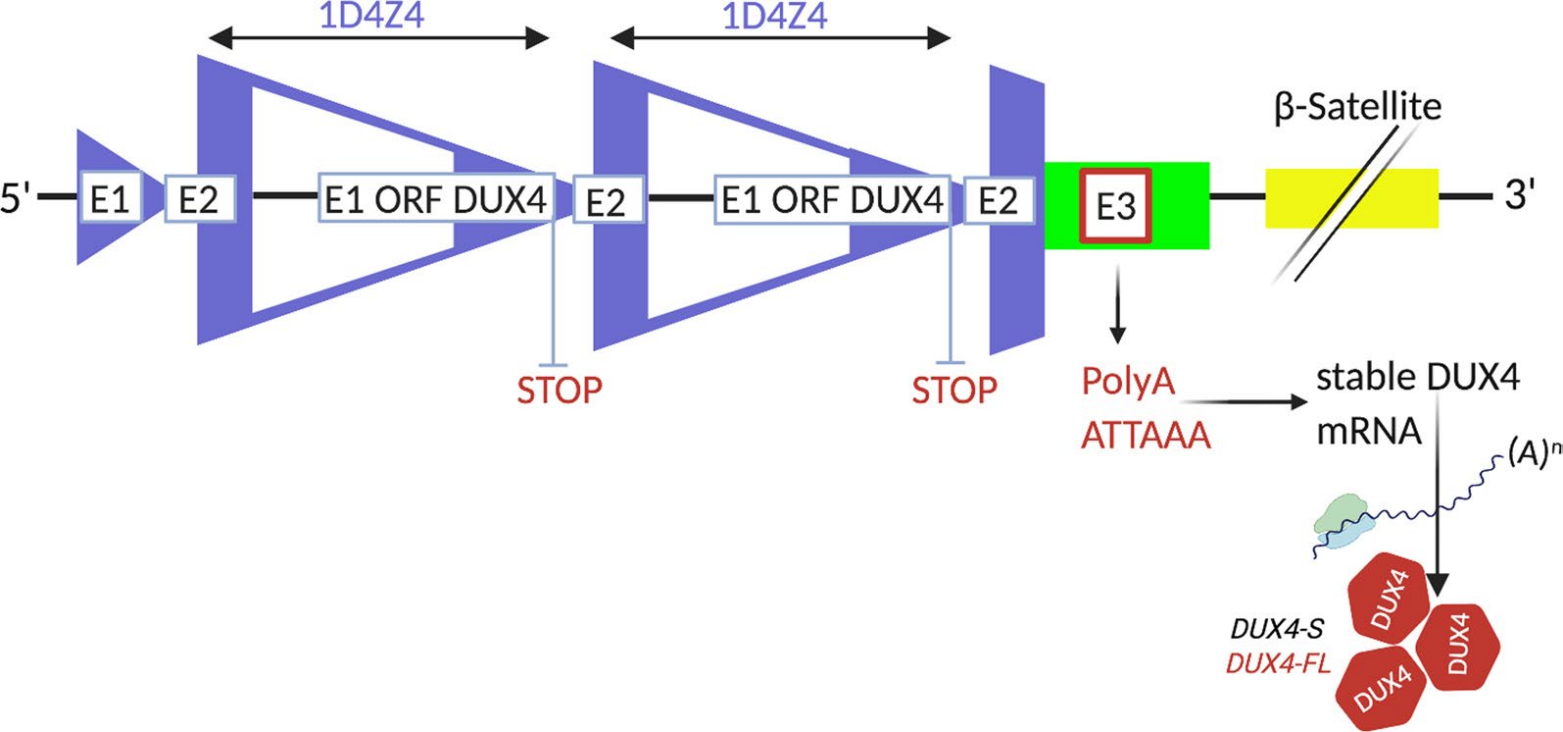
Requirements for *DUX4* gene expression. *DUX4* expression is only possible at the most distal D4Z4 repeat of the 4qA alleles. Exon 3, which is located outside the D4Z4 repeats in the pLAM1 region is carrying the variant *ATTAAA* that provides a PAS enabling the expression of the most distal copy of the *DUX4* gene into *DUX4* mRNA and then *DUX4* protein [39]. Whilst healthy individuals especially generate the non-toxic splice form of *DUX4-S* (encodes the amino-terminal part and both homeodomains; does not contain the C-terminal domain) [73], FSHD muscle produces toxic *DUX4-FL* mRNA [32]

In FSHD muscle only *DUX4* from the most distal repeat unit can be expressed in a stable manner. This is due to genetic elements downstream to the repeat that are necessary for mRNA processing [32, 78]. As illustrated in Fig. 4, the *DUX4-FL* gene is composed of three exons. While its open reading frame (ORF) is entirely incorporated in the first exon, exon 2 and 3 are non-coding regions (3′UTR) [39]. Besides, exon 3 is positioned outside of the D4Z4 repeats. On permissive chromosomes, in the last copy of the *DUX4-FL* gene the third exon stabilizes the transcript due to the presence of the PAS [26, 79].

Reverse Transcription Polymerase Chain Reaction (RT-PCR) and immunofluorescence studies showed a small number of myonuclei, which express relatively high levels of *DUX4-FL*. There is no uniform low expression level in all nuclei [32, 79] leading to difficulties in detecting its expression from patient samples when searching for the disease’s origin [72]. Interestingly, two isoforms of *DUX4-FL* were discovered as a result of alternative splicing events. Snider et al. (2010) showed the existence of *DUX4-FL* mRNA and the *DUX4-FL-3′* splice form in muscle biopsies of FSHD patients. Control muscle cells did not contain noticeable amounts of *DUX4-FL* mRNA. *DUX4-S* was expressed in all control samples with the SSLP 4A161 and in some of the FSHD samples. These data demonstrate that FSHD as well as control muscle cells “actively transcribe *DUX4*.” [32]. In the following paragraphs *DUX4-FL* will be referred to as *DUX4* because it is considered to cause FSHD.

According to Lim et al. (2020) “the FSHD phenotype may be the cumulative result of extensive aberrant signalling across time” [80] with unpredictable bursts of expression [81]. Since *DUX4* induces hundreds of different target genes, also affecting the induction of apoptosis [82] and atrophic muscle fibres [7] (see “DUX4 Downstream Signalling”)*,* its repetitive expression over years may lead to noticeable loss of the specific muscle areas (see Fig. 1). In this regard Mariot et al. (2015) suggested, that *FAT1* levels might determine which muscles will exhibit early and late disease onset, while *DUX4* may worsen the muscle phenotype [83] (see “FAT1 in the context of long distance interactions”). Whilst the nature of *DUX4* has been researched extensively and great knowledge has been gathered in the last decade, the context of genetic interaction regarding methylation levels is not fully understood and direct target genes, which bind to the *DUX4* promoter, are still not fully known*.* Nevertheless, molecular key features of *DUX4*, such as exon 3 which is specifically associated with the pathogenic *DUX4* transcript, could be revealed. This allows for promising treatment approaches in the context of future drug development (see “AOs” in “Molecular treatment strategies”).

### Methods of diagnosis

**Fig. 5:**
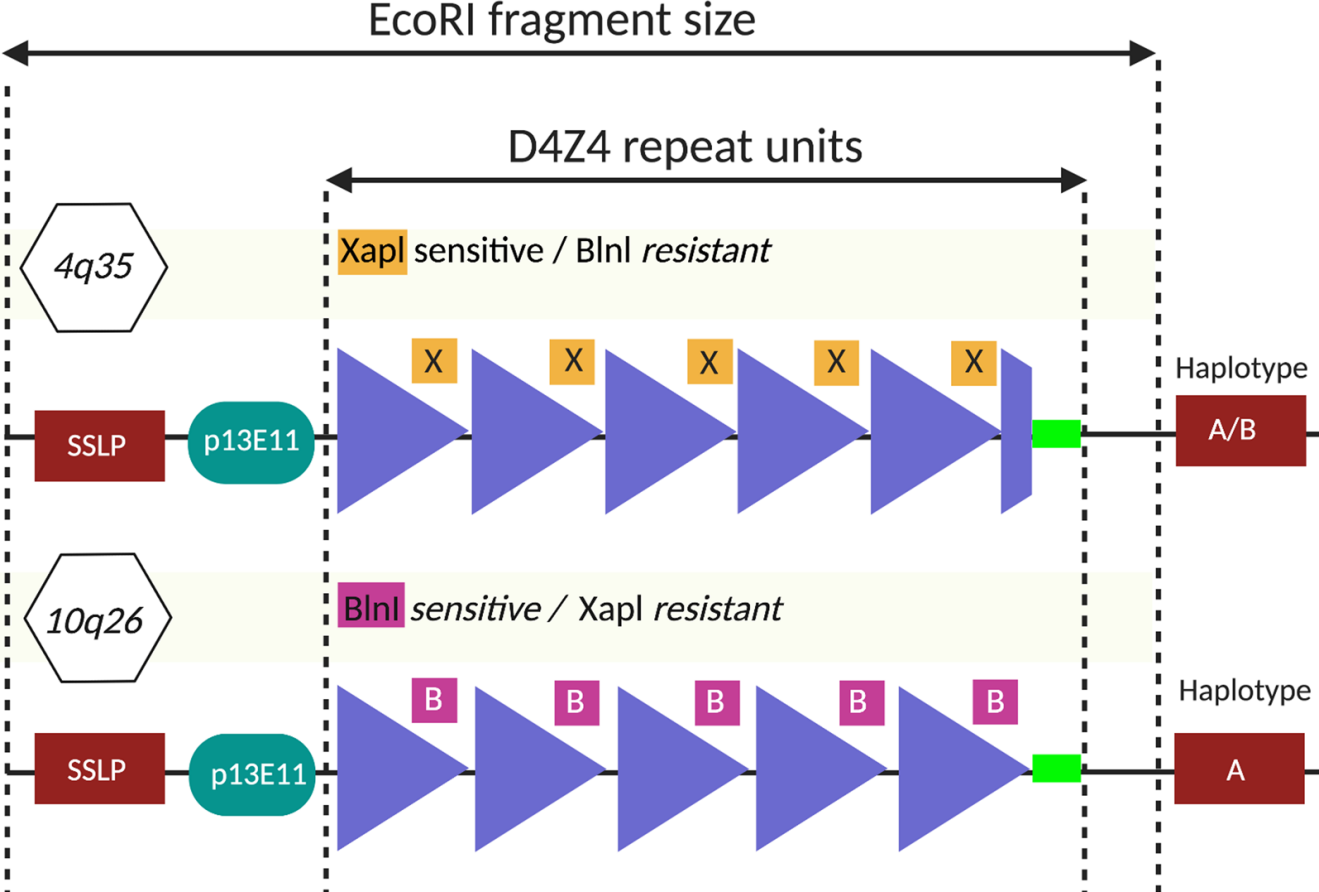
Schematic representation of the methods used for FSHD1 diagnosis. The D4Z4 repeat array is indicated with triangles (in dark blue). D4Z4 repeat units on chromosomes 4 and 10 can be separated because all repeats on 10q contain *Bln*I restriction sites (whereas all D4Z4 repeats on 4q contain *Xap*I restriction sites) [238]

Diagnosis of FSHD is commonly performed using Southern Blotting [84] with the blot hybridization probe p13E-11 [85]. Double enzyme digestion using *Eco*RI and *Bln*I—from the 4q35 and the 10q26 regions—is performed as the probe identifies two pairs of *Eco*RI alleles. As depicted in Fig. 5, 4q35 was found to be *Bln*I-resistant, whilst 10q26 is *Bln*I-sensitive [86–88]. *Eco*RI cuts at both ends thereby releasing complete D4Z4 repeat arrays with little flanking sequences. The *Eco*RI fragments from 10q26 are shortened to below the detection limit due to *Bln*I [84]. Another restriction enzyme—called *Xap*I—is also used for diagnosis of FSHD. In contrast to *Bln*I, *Xap*I leaves chromosome 10-type units undigested when fragmenting chromosome 4-type D4Z4 units [36].

Both alleles of the 4q35 locus are examined whilst observing the molecular results from EDTA blood. One allele is typically normal-sized compared to healthy individuals [89], whilst the other allele contains fragments (≤ 43 kb) after *Eco*RI cleavage and fragments (≤ 40 kb) after *Eco*RI and *Bln*I double digestion. Morover, fragments associated with FSHD can no longer be detected after *Xap*I cleavage. Eight and nine D4Z4 repeats (32–35 kb *Eco*RI fragment size) are currently defined as disease-associated [90–92]. Other researchers point out that 10–11 repeats (38–41 kb) are a “grey-zone” [93] or “borderline” [94]. However, there is consensus that repeat numbers above 12 (≥ 45 kb) are beyond the diagnostic range for FSHD1 [92, 93].

The determined *Eco*RI fragment size provides information about the approximate repeat number at the locus as each D4Z4 repeat of chromosome 4q35 is approximately 3.3 kb long. Approximate D4Z4 repeat numbers can be determined from *Eco*RI-fragment sizes by using the formula [93, 95, 96]:

$$Repeats= \frac{EcoRi~fragment~size~in~kb-5kb~flanking~sequence }{3.3kb}$$

For FSHD2 diagnosis, methylation analysis of the D4Z4 region and *SMCHD1* sequencing on chromosome 18 is performed. This is done only if chromosome 4 also offers the “permissive” 4qA allele [55, 97] as FSHD2 is inherited in a digenic manner [45].

The traditional genetic FSHD diagnosis method is labour-intensive, requires a lot of time, and needs a large quantity of high-quality DNA. Therefore, whole genome optical mapping is now commonly used—especially for prenatal diagnosis. In this regard, Zheng et al. (2019) showed that Bionano optical mapping can identify the number of D4Z4 repeats and avoid interference of the 10q26 homologous region. Moreover, whole genome optical mapping combined with karyomapping results in a quick and precise diagnosis of FSHD1. Whilst the Southern Blot-method is only able to estimate the number of D4Z4 units, whole genome optical mapping is faster and more accurate [89]. However, the whole genome optical mapping technique also shows some insufficiencies as the estimated proportion of mosaicism might be less precise when several alleles differ significantly in the number of repeats [98].

Molecular combing to directly visualize allelic combinations correlating with FSHD—by identifying “somatic mosaicism, 4q-10q translocations, p13E-11 deletion, and other non-canonical modifications” [99]—has been developed for more complex disease variations [100]. Southern Blot alone may be insufficient for interpreting results in instances where translocations have been shown in both healthy individuals and FSHD patients [101]. 4qA-10qA translocations may arise and 10qB alleles have been reported as result from the translocation of 4qB alleles [44] due to the high level of sequence homology between chromosome 4q and 10q, which facilitates inter-chromosomal exchanges [87]. Common Southern Blot is usually the first method used for the diagnosis of patients presenting FSHD phenotype, followed by Molecular Combing—which can be performed to specify results [99].

### Disease monitoring

Regular neurologist or physiatrist visits help monitor the progress of FSHD, especially as there can be deficits in respiratory function over a long period of time without any severe symptoms. Furthermore, respiratory compensatory mechanisms can allow the organism to adapt to increasing amounts of carbon dioxide (CO_2_) in the blood during the night due to sleep-related hypoventilation. This is known as hypercarbia (hypercapnia) [102, 103], and has been reported to cause symptoms that patients may become tolerant of, such as morning headaches, cognitive difficulties, or daytime fatigue [104]. In more severe cases when gas exchange worsens due to deficits in respiratory function, an early support with non-invasive ventilation (NIV) must be considered as there is a risk of acute respiratory failure [103].

Some early-onset patients may have “moderate to profound bilateral sensorineural hearing loss and sight-threatening retinal abnormalities “[105] (Coats’ Syndrome [106]). In this case, it is essential to regularly check hearing and vision [105]. FSHD is a slowly progressive disease, making it difficult to evaluate modifications which may be triggered by relatively short-term treatment approaches. Therefore sensitive prognostic biomarkers are highly valuable for clinical trial design. Magnetic resonance imaging (MRI) is one technique that also indicates an early phase of muscle damage. MRI recognizes muscle injury by increased *signal on short-tau inversion recovery* (STIR) sequences demonstrating oedema/inflammation which anticipates fatty replacement of single muscles. This is consistent with the suggested model of disease pathophysiology which states that bursts of *DUX4* expression initiate a cascade of downstream events, also possibly encompassing an inflammatory-immune response (see “DUX4 Downstream Signalling”)*.* Against this background, STIR positive (STIR +) muscle lesions have been suggested as biomarkers regarding disease activity. According to Monforte et al. (2019) examination of patients with STIR + muscles—who are thus more exposed to disease progression in a short period of time—would strongly improve the chance to discover a considerable impact of an investigational treatment. Furthermore, a higher amount of STIR + muscles at baseline was shown to anticipate deterioration at follow-up studies. This confirms the association between STIR + lesions and disease progression [107].

Electrical impedance myography (EIM) is an alternative tool that measures changes in muscle. EIM uses electrical current to identify the impedance to current flow through a specific muscle or muscle group [108]. EIM has been used in several neuromuscular diseases—including FSHD [109]—indicating its reliability [109–113]. According to LoRusso et al. (2019) EIM is “painless, requires minimal training, and does not require specific expertise in post-processing” [108]. Nevertheless, in a later study encompassing 32 patients EIM did not detect meaningful disease progression over one year in a clinically stable group of patients [108]. According to Mul et al. (2018), it is unclear whether this is because of technological limitations or the slow disease progression in that cohort of FSHD patients during the specific time frame [110].

### Trial readiness

Apart from manual muscle testing (MMT) and quantitative myometry (QMT), there are no approved outcome measures which can be consistently used in clinical trials. Besides, individual functional measures may not be sensitive to disease progression over 12 months [108]. A large, international, multi-centre prospective study was therefore initiated in March 2018 in order to improve clinical trial tools and methodology in the context of drug development for FSHD [108]. The estimated completion date of the study is March 2022 (clinicaltrials.gov: NCT03458832) [114]. The 18-month long study includes 220 FSHD patients from the United States and Europe. The primary goal is to “hasten drug development for FSHD by validating two novel clinical outcome assessments (COAs) and refining clinical trial strategies” [108]. Novel COAs are the functional *FSHD composite outcome measure* FSHD-COM and the skeletal muscle biomarker EIM (see “Disease monitoring”)*.* FSHD-COM encompasses 18 evaluator-administered motor tasks in the areas of shoulder/arm, hand, core/abdominal, leg, and balance function. Reliability and sensitivity to disease progression are yet to be proved [108, 115]. The focus is set on the evaluation of the “test–retest reliability, validity, and sensitivity to disease progression, and minimal clinically important changes” of the new COAs [108]. Visits are at baseline and at months 3, 12, and 18. Statistical methods will be further implemented at each point to specify subgroups, likely to progress over 12–18 months in varying degrees. The aim of the study is to analyse links between demographic, genetic factors and disease progression in order to refine eligibility criteria for future clinical trials [108].

### *FAT1* in the context of long distance interactions

**Fig. 6:**
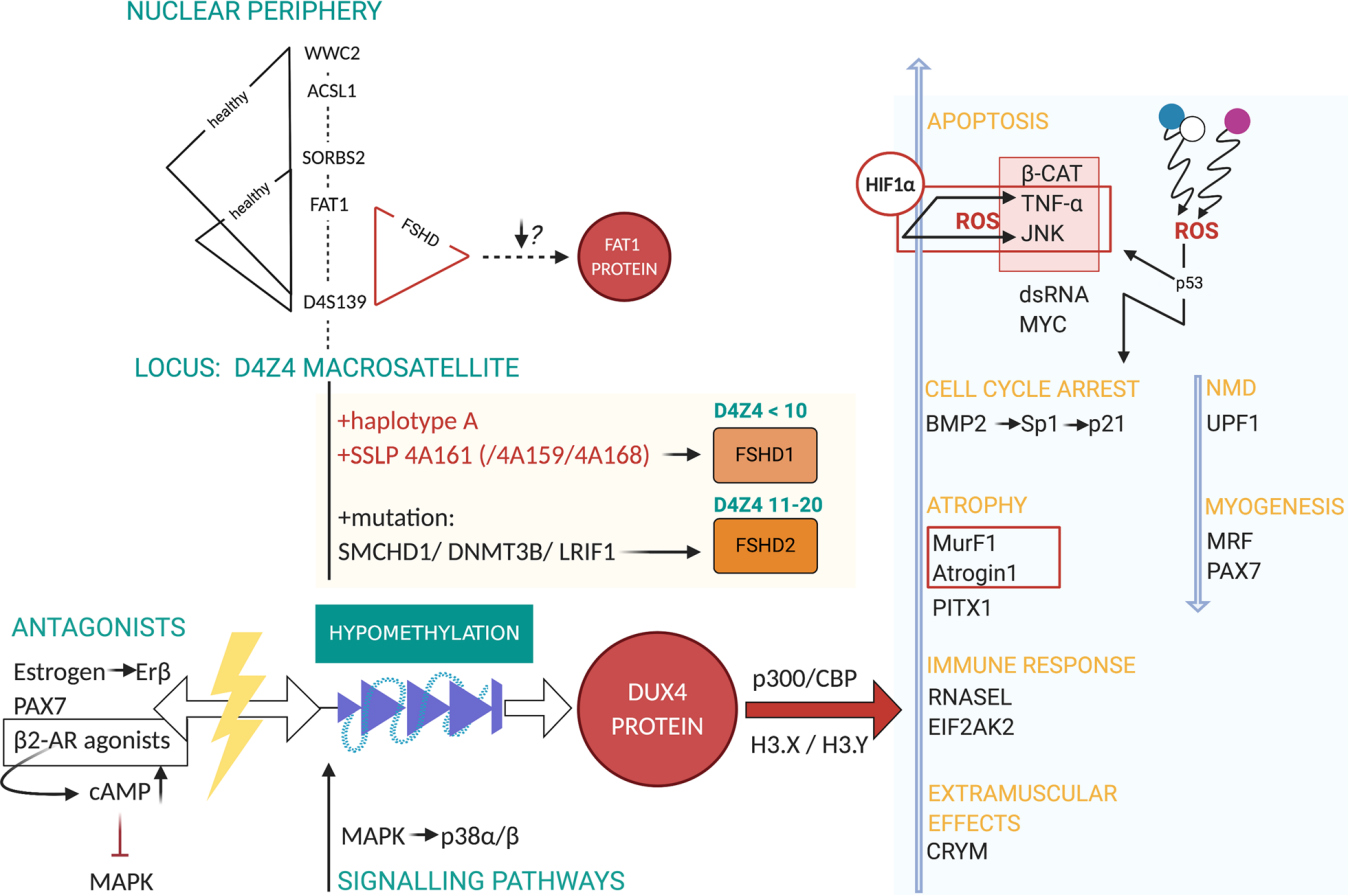
Overview of *DUX4* signalling. Antagonists of *DUX4* are depicted on the left; Signalling pathways for *DUX4* gene activation have been associated with MAPK, especially p38α/β, bottom); *DUX4* is located within the D4Z4 macrosatellite, which interacts with subtelomeric regions and the nuclear lamina via long distance-interactions, influencing (1) methylation levels and (2) gene expression. Interactions found in control cells and in FSHD are depicted at the top of the graphic. (For reasons of clarity, interactions of FSHD2 cells are not illustrated here, but are mentioned in the text.) *FAT1* gene is shown to aggravate FSHD, when expressed at low levels. Muscles are more sensitive to gene deregulations and become affected earlier [83]; *DUX4* generally requires help of *p300/CBP* and *H3.X/H3.Y* for target gene expression; Depicted on the right: *DUX4* expression affects multiple genes, which are either upregulated or downregulated by *DUX4* protein. ROS seem to have a prominent role in disease mechanism and are probably activated directly by *DUX4* itself and indirectly through further targets of *DUX4*

FSHD is known to affect specific muscles whilst other muscles are spared [83]. In this regard, *FAT Atypical Cadherin 1* (*FAT1*) has been found to affect muscle morphogenesis as it influences the shape of subsets of face and shoulder muscles, partly by polarizing the direction of collectively migrating myoblasts [116]. Mariot et al. (2015) demonstrated that levels of *FAT1* are lower in muscles that are affected at early stages of FSHD progression than in healthy muscles or muscles that are affected later in time. Furthermore, the authors demonstrated that *FAT1* expression is independent of *DUX4* [83]. The locus of chromosome 4 might therefore be particularly important as (in contrast to chromosome 10) the telomere of 4q is localized at the nuclear envelope. Intriguingly, mediation of interaction with the nuclear envelope was associated with genomic regions proximal to the D4Z4 repeat, such as D4S139, a variable number tandem repeat (VNTR) locus [117] that also interacts with *FAT1*. Gaillard et al. (2019) showed the existence of functional long distance-interactions between D4Z4, the nuclear lamina, and the telomere by using three-dimensional Fluorescent In Situ Hybridization (3D FISH). According to the authors, the 4q35 locus encompasses two topologically associating domains (ToADs) encompassing four domains connected with the nuclear lamina (Lamin Associated Domains, LADs), with each one overlapping with different gene areas. The most proximate LAD to D4139 overlaps with an area encompassing the *FAT1* gene. It was shown that the mean distance between the D4S139 region and *FAT1* was particularly higher in control cells than in FSHD cells, and that interaction of D4S139-*FAT1* was restricted to FSHD. To illustrate these findings, Fig. 6 shows the different interactions found in control cells and in FSHD cells. There is an overall loss of interaction with other gene areas in the case of FSHD. (The data also demonstrated the involvement of other genes as well. For detailed information see Gaillard et al. 2019.) However, in the case of FSHD2 and the non-contracted allele of FSHD1, interactions between *FAT1* and *Sorbin and SH3 domain-containing protein 2* (*SORBS2*) could be detected further. The loss of interaction regarding the contracted FSHD1 allele [60] might contribute to FSHD1 pathogenesis as *SORBS2* could be, inter alia, detected at the Z-line in skeletal muscle [118].

As D4Z4 is reported to act as transcriptional repressor CTCF—which organizes long distance-interactions between the telomere—subtelomeric regions and the nuclear lamina by distance-dependant mechanisms, it could be hypothesized that decreased levels in *FAT1* expression might be the result of a changed communication between the involved genes. If so, further questions—including,*“When does FAT1 expression level trigger FSHD? Is it during fetal development, after birth, or both?”* [83]—as in the case of *DUX4* may be answered as “both” because interaction has been shown in muscle biopsies from foetuses as well as adult individuals [60]. However, the type of interaction/ the direct impacts of interaction on protein expression have not been explored further.

### *DUX4* downstream signalling

When reactivated in skeletal muscle, the early embryonic program of *DUX4* [69] activates several hundreds of target genes (e.g. *ZSCAN4*, *MBD3L2*, *TRIM43*… [28, 119]) and initiates numerous detrimental events including the activation of the inflammatory immune response [120], the induction of apoptosis [82], atrophic muscle fibres [7], oxidative stress [121], and an altered muscle cell differentiation in myogenesis [122]. Affected FSHD muscles show fibro-fatty replacement [123, 124]. It is still unclear if inflammation displays a protective effort against the *DUX4-*driven damaging effects or a direct deleterious component of the *DUX4* cascade [107]. Most findings described in this paragraph have been obtained in in vitro cell models or in transgenic mice overexpressing *DUX4*. Figure 6 shows an summary of the literature findings of *DUX4* pathogenesis. Also *FAT1* is depicted as it is reported to influence disease severity [83].

#### β-catenin

Whilst Block et al. (2013) showed that activation of the *Wnt/β-catenin* signalling pathway suppressed *DUX4* transcription [125], Banerji et al. (2014) identified *β-catenin* as the “main coordinator of FSHD-associated protein interaction signalling” [126]. Pathways encompassing *HIF1-α* (see “DUX4 downstream signalling: HIF1-α”), *tumor necrosis factor* (*TNF*)*-α* and *c-Jun N-terminal kinases* (*JNK*) were shown to be clearly disturbed [126] by *β-catenin*. The latter are involved in oxidative stress-induced cell death (see “DUX4 Downstream Signalling: ROS”), revealing that *β-catenin* is highly influencing *DUX4*-mediated toxicity. According to Lim et al. (2020), “this consequently results in a negative feedback loop wherein DUX4 activates *Wnt/β-catenin *signalling, which represses its own expression.” This may be the reason for the low amount of detectable *DUX4* nuclei in FSHD muscle cells [80].

#### dsRNA, RNASEL and EIF2AK2

Shadle et al. (2017) found that *DUX4* expression increased nuclear double-stranded RNA (dsRNA) accumulation. This can trigger a signalling cascade that inhibits translation and induces apoptosis. The appearance of dsRNAs activates expression of *RNASEL and EIF2AK2*, which are effectors of the innate immune response particularly against viral invasion by either cleaving intruding RNAs or inhibiting translation [82].

#### P53 vs. BMP2

While Wallace et al. (2011) suggested that *DUX4*-induced cell death relies on the *p53* pathway [127], other study findings doubt that *p53* is a direct consequence of *DUX4* expression [128]. Interestingly, *DUX4* overexpression in vitro showed *Cyclin Dependent Kinase Inhibitor 1A* (*CDKN1A*) expression, a major *p53* target, which codes for *p21*. However, studies that observed this did not show upregulation of *p53* [129, 130], but knockdown of *CDKN1A* was shown to enhance the proliferative capacity of *DUX4-*transfected cells. In this context, it could be shown in vivo that *DUX4*-induces binding of *specificity protein 1* (*sp1*) to *p21* promoter [129]. According to Xu et al. (2014) *DUX4* may activate *CDKN1A* expression through the *bone morphogenetic protein* (*BMP*)*-2* signalling pathway, which is an upstream actor of *Sp1.* According to the authors, *BMP2* mRNA increased after enforced *DUX4* expression and was accompanied with an increase of *Sp1* and *p21*.

Apart from this, *p53* might be triggered by other events as it is reported to correlate with Reactive Oxygen Species (ROS) by acting either as an antioxidant or a pro-oxidant regulating redox homeostasis. *p53* is described to either reduce ROS levels or to induce cell cycle arrest, senescence, and apoptosis [131].

#### MYC

*DUX4* upregulates *MYC,* which functions in cell cycle progression and as a mediator of extrinsic and intrinsic pathways of apoptosis. *DUX4* overexpression enhances *MYC*-mediated cell death by stabilizing *MYC* mRNA [82].

#### Estrogen

Teveroni et al. (2017) suggested that the reason why females tend to be less severely affected by FSHD than males is due to estrogens counteracting the differentiation impairment of FSHD myoblasts by *estrogen receptor β* (*ERβ*). While they observed an enhanced recruitment of *DUX4* transcription factor in the nucleus during muscle differentiation, *ERβ* intervened against this recruitment by returning *DUX4* into the cytoplasm [132]. However, according to recent clinical findings, differences in endogenous estrogen exposure during life did not appear to have a clinically relevant modifying impact on disease severity in female patients. It suggests that additional sex-related aspects might also play a role in the further protection from *DUX4*-induced muscle damage [133].

#### β2-AR

**Fig. 7:**
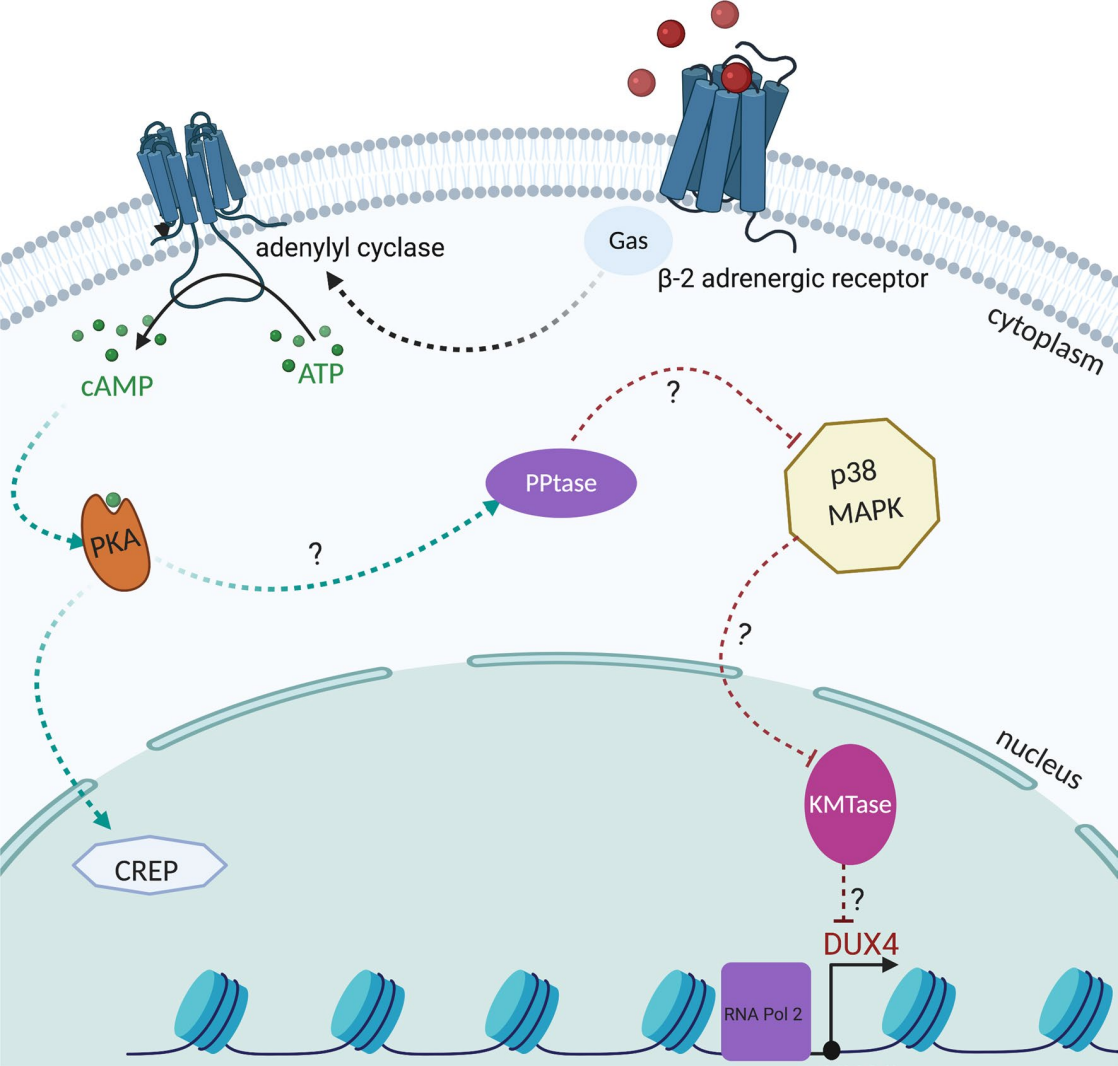
Model of *DUX4* regulation by β-2 adrenergic signalling. β-2AR binding of agonists induces G protein-mediated activation of adenylyl cyclase, which subsequently catalyzes the formation of cAMP. Downstream effectors of cAMP are PKA-dependent and PKA-independent pathways. β-2 agonist-mediated inhibition effects on *DUX4* expression are likely mediated through PKA-independent pathways acting through signalling molecules such as phosphatases (PPtases) and MAPKs to effect chromatin modifiers, e.g. lysine methyltransferases (KMTases), to influence transcription of the *DUX4* gene [140]

Prior to the discovery of β2-adrenergic receptor (β2-AR)-agonists influencing FSHD pathogenesis, several trials with β2-AR like clenbuterol or salbutamol had been conducted due to their anabolic effects [134–136]. Although they were tried as anabolic agents, they appear to have a direct effect on a *DUX4-*induced mechanism. While further trials in FSHD patients with salbutamol have shown no major impact as a routine treatment for FSHD [18, 137–139], investigations on β2-AR have revealed an involvement in the regulation of the D4Z4 array in somatic cells [140]. The participation of molecules and the interconnection between different pathways is still not fully understood [141]. β2-AR-agonists increase cAMP levels via adenylate cyclase stimulation through trimeric Gs proteins (see Fig. 7). Efforts to further explore this signalling pathway led to the identification of p38 mitogen activated protein kinase (MAPK) as a major regulator of *DUX4* expression. In vitro experiments demonstrated that clinically advanced p38α or p38β inhibitors are able to suppress *DUX4* expression in FSHD myoblasts and differentiating myocytes. This demonstrated that each of these kinase isoforms plays a different role in activating *DUX4*. It was shown that p38 inhibitors successfully suppressed *DUX4* expression in a mouse xenograft model of human FSHD gene regulation (see “Therapeutic approaches”) [34, 141]*.*

#### MRF

*DUX4* expressed at high levels causes rapid cell death, but at low levels it interferes with master myogenic transcription factors—predominantly with *MyoD.* The latter is part of the myogenic regulatory factor (MRF) family, which also comprises the muscle specific proteins *Myf5, myogenin* (*MyoG*), and *MRF4* (*Myf6*) [122, 142]. (Several reviews and textbooks regarding the different steps of myogenesis are available.) Whilst *Myf5* mRNA was shown to be upregulated by *DUX4* possibly representing a compensatory mechanism, Knopp et al. (2016), who analyzed transgenic mice carrying a human D4Z4 genomic locus from an FSHD-affected individual, showed that *DUX4* inhibits both *MyoD* and *MyoG* gene expression to produce a differentiation defect that cannot be controlled by upregulation of *Myf5. DUX4* and its transcriptional activity can be discovered in differentiating human myoblasts [32, 78, 143–145]. There is a “maintenance of a stem-cell-like and less-differentiated state” [143] due to *DUX4* expression.

#### Atrogin1 and MuRF1

*DUX4* induces the expression of the muscle-specific E3 ubiquitin ligases *Atrogin1* (*MAFbx*) and *MuRF1*. The ubiquitin proteasome pathway plays a major role in regulating protein degradation and muscle atrophy [146, 147]. Lagirand-Cantaloube et al. (2009) further showed that *Atrogin1* suppresses *MyoD* specific transcriptional activity among the muscle fibre [148].

#### PAX7

Whilst *PAX3* influences early skeletal muscle formation in the embryo, *PAX7* dominates during post-natal growth and muscle regeneration in adult individuals [149]. In FSHD skeletal muscle *PAX7* target genes are globally repressed [150]. The paired-homeobox transcription factor stimulates proliferation whilst inhibiting differentiation [151], thus regulating the expansion of satellite cells during myogenesis [149, 152]. Studies in mice showed that *PAX7* could prevent *DUX4-*mediated toxicity in a dose-dependent way and re-establish myogenic gene expression [130]. While Bosnakovski et al. (2017) suggested some type of competitive interaction [122], Haynes et al. (2017) observed that nuclei do not express both *DUX4* and *PAX7* proteins and that the transcription factors may not compete for the same genomic binding sites [153]. Remarkably, “*DUX4* and *PAX7* homeodomains show 100% identity in their DNA-binding amino acids” [80]. *PAX7* target gene repression is a significant biomarker as it correlates with disease severity independently of *DUX4* [154].

#### UPF1

The evolutionarily conserved protein *Up-frameshift protein 1* (*UPF1*) is one of the main effectors of nonsense-mediated mRNA decay (NMD) [155]. Feng et al. (2015) reported that *DUX4* stimulates the degradation of *UPF1*, inducing global accumulation of RNAs, usually degraded as NMD substrates [156]. Interestingly, *DUX4* mRNA itself is degraded by NMD. Therefore, “inhibition of NMD by *DUX4* protein stabilizes *DUX4* mRNA through a double-negative feedback loop in FSHD muscle.” [157]

#### CRYM

Vanderplanck et al. (2011) showed that *DUX4* induces *CRYM* (*μ-Crystallin*) by direct promoter activation. The thyroid-hormone binding protein with nicotinamide adenine dinucleotide phosphate (NADPH)-dependent activity influences differentiation and oxidative stress responses [146, 158]. The physiologic function in skeletal muscle remains to be elucidated. Interestingly, it is additionally expressed in the cochlea and vestibule of the inner ear [159], potentially explaining the occurrence of retinal abnormalities [160] and high-frequency hearing loss [161] in some FSHD patients with severe phenotype.

#### PITX1

The *paired-like homeodomain transcription factor 1*, which encodes a transcription factor that is the “master switch for hindlimb development in embryogenesis” [35], is particularly triggered in FSHD muscles as compared to 11 neuromuscular disorders [7]. It has been discussed as direct *DUX4* target gene [78, 127]. However, according to Zhang et al. (2016) it does not interact physically or functionally with the *PITX1* promoter sequence as there is no optimal CT motif [77] (see “DUX4 Protein”)*. PITX1* has been suggested to explain the asymmetric involvement of FSHD muscles due to its role in creating left–right asymmetry [80]. It is still not well understood how *PITX1* contributes to FSHD pathogenesis [7] apart from causing atrophy in adult skeletal muscles and its involvement in inflammation.

#### H3.X and H3.Y

Resnick et al. (2019) showed that *DUX4* induces expression of the histone variants *H3.X* and *H3.Y*. Following a brief pulse of *DUX4*, these histones incorporate into genes transcriptionally induced by *DUX4* and contribute to higher persistence and to improved reactivation of *DUX4* target gene expression [162].

#### P300

Svensson et al. (2020) demonstrated a general requirement for *p300* including *CREB-binding protein* (*CBP*) in skeletal muscle contractile function, transcriptional homeostasis, and organism survival [163]*. DUX4* protein also interacts with the transcriptional coactivator *p300* and utilizes its acetyltransferase activity to induce expression of many of its target genes [34, 75, 164].

#### ROS

Several indices for the accumulation of ROS in FSHD muscle have been reported. For example, Dimitriev et al. (2016) showed constitutive DNA damage in cultured myoblasts from FSHD, which could be diminished by addition of an antioxidant [121]. Furthermore, several markers for increased oxidative stress, including 4-hydroxynonenal-modified proteins, protein carbonylation, and a lower glutathion (GSH)/ glutathione disulfide (GSSG) ratio have been found in FSHD muscle biopsies [165]. Importantly, *DUX4* was shown to reduce the expression of several glutathione redox pathway-associated genes—including phospholipid hydroperoxide glutathione peroxidase (GPX4), glutathione S-transferase A4 (GSTA4) and glutathione S-transferase omega-1 (GSTO1)—in a *DUX4-*inducible myoblast in vitro model [130]. In muscle biopsies of FSHD patients, however, higher levels of glutathione S-transferase, superoxide dismutase (SOD), catalase, and glutathione reductase have been found—contradicting the aforementioned in vitro results [165, 166]. In this regard, *p53* and its performance as an antioxidant or pro-oxidant under different stress levels could be taken into account. Whereas the antioxidant function of *p53* is particularly attained via upregulating the typical antioxidant enzymes, the procedures by which *p53* increases ROS are less understood (see “DUX4 Downstream Signalling: P53 vs. BMP2”) [131]. Apart from this, it is currently hypothesised, that increased expression of antioxidant defence system proteins is a result of compensatory mechanisms, activated by prolonged and increased oxidative stress [167]. The exact origin of increased ROS, however, still remains unclear. Mitochondrial dysfunction is currently discussed as major source for increased ROS, resulting from decreased oxygen storage capacity [167]. However, muscle cells from FSHD patients are generally acknowledged to have an increased susceptibility to oxidative stress, also emphasizing that mechanisms generating small amounts of ROS should be taken into consideration [165–168]. Increased lactate concentrations, for example, are known to induce low amounts of ROS [169]. Under physiological conditions, this low increase in ROS results in an increased antioxidant defence system or can induce erythroid differentiation [170, 171]. In FSHD muscle, an increase of lactate is evident, as an increased expression of lactate dehydrogenase has been observed in vitro and in vivo [172, 173]. In line, lower levels of myoglobin and higher activity of *HIF1-α* strongly indicate a lower oxygen storage capacity in combination with increased anaerobic production of lactate [126, 172, 174, 175]. Therefore, higher basal ROS levels may be partially caused by increased lactate levels. However, this hypothesis awaits further confirmation by future studies. Also, it should be noted that the release of interleukin (IL)-6, a central mediator of inflammation, can be induced by ROS [176, 177]. Therefore, the extensive inflammation reported in FSHD muscle could be induced by an initial increase in ROS [167, 178]. As several types of immune cells are known to produce ROS at the site of inflammation, this may result—in combination with the perturbed antioxidative defence system in myocytes—in a chronic inflammation that is held constant via a paracrine mechanism. This may further result in a fatal loop [121] as increased ROS was also identified as the central mediator in the formation of atrophic myotubes.

#### HIF1-α

Lek et al. (2020) conducted an unbiased screen utilizing a genome-wide CRISPR-Cas9 loss-of-function library in order to detect possible targets that influence *DUX4-*mediated cell death. Focus was set on genes, in which loss-of-function contributed to survival of muscle cells when *DUX4* was expressed. A pathogenic association to the cellular hypoxia response was shown. This was found to be the main driver of *DUX4-*induced cell death. Under hypoxic conditions, *hypoxia-inducible factor 1* (*HIF1*)-*α* is stabilized and transfers into the nucleus. It dimerizes with *ARNT* and shapes the *HIF* transcription factor. *HIF*s—in combination with the coactivators *CBP* and *p300*—mediate transcription of hypoxia response genes and *CDKN1A* (one of its target genes). This combination is reported to mediate hypoxia-related growth arrest [179].

### Animal models

The D4Z4 macrosatellite encoding the *DUX4* retrogene is “specific to old world primates” [180, 181]. This negates the possibility of working with a “natural” model of the disease in commonly used laboratory animal species. Modelling FSHD in non-primate species that do not express endogenous *DUX4* raises concerns of whether the same downstream gene targets and regulatory networks exist, and can be activated as a consequence of *DUX4* misexpression to cause disease as in primates [180].

In cultured human FSHD muscle cells there are bursts of *DUX4* expression from only a minority of myonuclei [162]. Moreover, protein has not been found directly in patient biopsies. Approaches to model *DUX4* myopathy in mice have proven to be too cytotoxic, resulting in embryonic lethality, or in lacking muscle phenotypes [182]. The majority of current laboratory animal models of FSHD try to mimic *DUX4* misexpression via the transgenic insertion or injection of *DUX4.* An example of this would be *FLExDUX4*—which is a line of conditional floxed *DUX4-FL* transgenic mice—that was developed by Jones and Jones (2018) in order to overcome “developmental toxicity of low *DUX4* expression from leaky transgenes” and create the conditions for *DUX4* animal experiments on mice that are viable and fertile (see: “Therapeutic approaches: AOs”) [120]*.* Also, other promising mouse models have been introduced, such as the doxycycline-inducible model iDUX4pA (pa for PAS) of Bosnakovski et al. (2017)—which makes *DUX4* expression dependent on its endogenous relatively inefficient PAS [182] or the tamoxifen-inducible (TIC)-*DUX4* mouse model of Giesige et al. (2018)—which conditionally expresses *DUX4* in muscles after tamoxifen injection [183]. Mueller et al. (2020) developed a promisingly accurate primate analogue model of FSHD by generating a procedure to xenograft immortalized human muscle precursor cells from FSHD patients into immunodeficient mice to create human muscle xenografts. They reported that FSHD cells “mature into well-organized and innervated human muscle fibres with minimal contamination of murine myonuclei” [184]. They were also managed to reconstitute the satellite cell niche within the xenografts. The xenografts are reported to be structurally comparable to intact human skeletal muscle as human myofibers are innervated and associate with human satellite cells [184]. Nevertheless, the host’s immune system must not reject tissue or cells from the donor for cross-species transplantation to be successful. X-irradiation is usually used to disrupt the host satellite cell niche for studies that aim to avoid host-derived muscle regeneration, whilst myotoxins or mechanical injury is used to destroy host muscle fibres [185]. Therefore, due to the use of immune-compromised mice, one disadvantage of the procedure would be the lack of information regarding the contribution of the immune system to disease and disease progression [186, 187].

According to Huml et al. (2020)—who compared literature findings regarding several animals with disease phenotype—there is no single, ideal model that can currently be used to wholly represent FSHD [4]. There are published models in mice, zebrafish, and dogs—each engineered through a different approach and producing different results [180]. Therefore, it is suggested that stakeholders should give high priority to collaborating to commit resources to developing an all-encompassing model for FSHD—inclusive of most muscle phenotypes—in one animal [4]. It may then be useful to analyse and use comparable parameters within different organisms. Hendrickson et al. (2017) showed that *DUX4* and its mouse ortholog, *DUX*, share central roles in cleavage-specific gene expression and a partial overlap of regulated genes. Determination of the extent of similarity in their transcriptional programs might provide more information about the design of mouse models for FSHD [28].

### Molecular treatment strategies

**Fig. 8:**
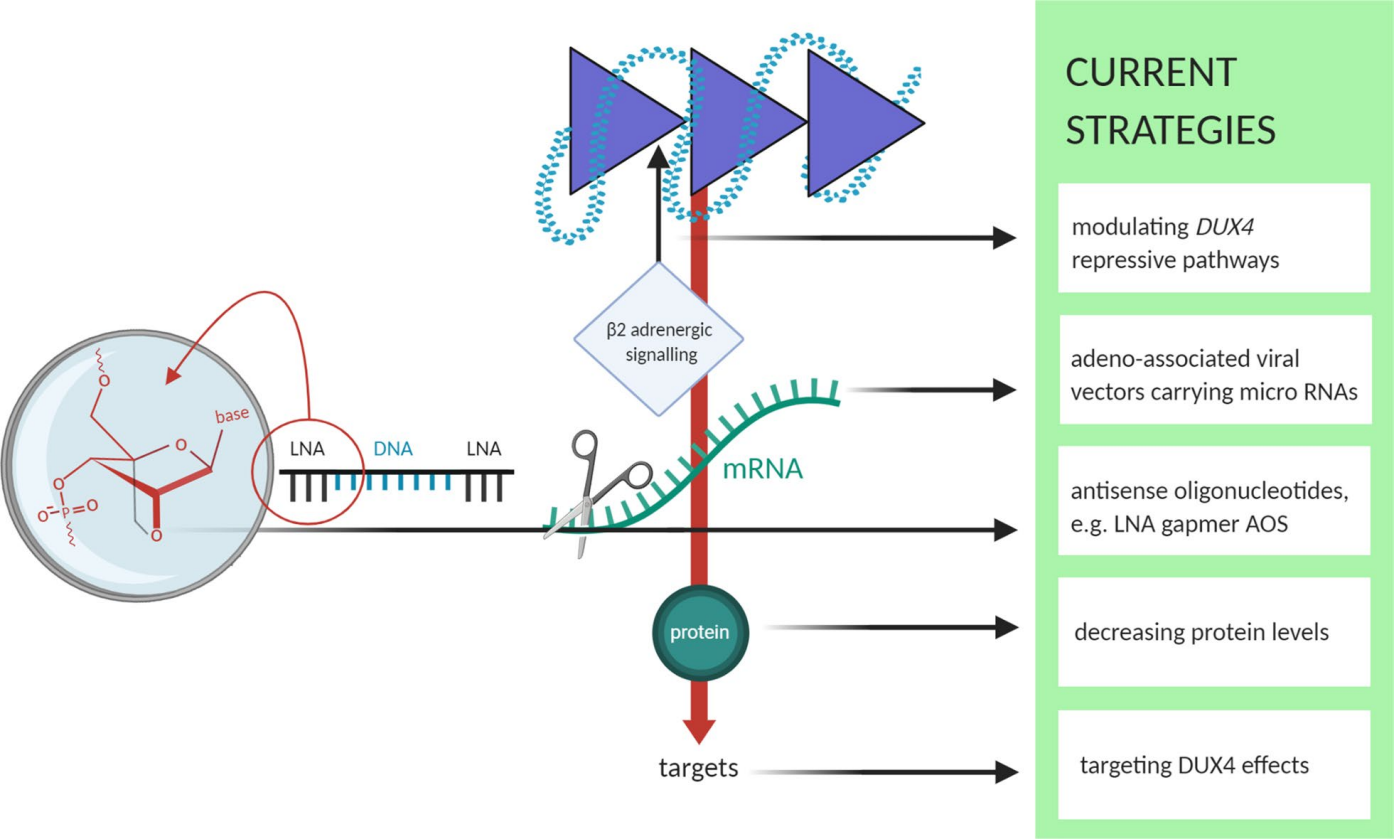
Current approaches for targeted treatment: modulating *DUX4* repressive pathways, targeting *DUX4* mRNA, *DUX4* protein, or cellular downstream effects of *DUX4* expression. Regarding *DUX4* mRNA LNA gapmer AOs are further illustrated on the right; These are single-stranded, short oligonucleotides containing a DNA portion flanked by LNA. The LNA parts increase the affinity for the target and provide nuclease resistance, DNA parts activate RNase H [207, 240, 241]; The extra methylene group of LNAs is attached between the 2′-O-and the 4′-positions “locks” the ribofuranosyl-ring in its 3′-endo conformation (shown under the magnifying glass) [240]

Current molecular treatment strategies are illustrated in Fig. 8 and include modulating *DUX4* repressive pathways, or targeting *DUX4* mRNA, *DUX4* protein, or cellular downstream effects of *DUX4* expression.

#### p38 inhibition

Efforts to further explore the signalling pathway of β2-adrenergic receptor*-*agonists led to the identification of p38 MAPK as a major regulator of *DUX4* expression (see “DUX4 Downstream Signalling: β2-AR”) [34, 141]. The four isoforms of p38 (α/β/γ/δ) are strongly triggered by several types of environmental stress and inflammatory cytokines, including “oxidative stress, UV irradiation, hypoxia, ischemia, *IL-1*, and *TNF-α*” [188]. P38α seems to be the main p38 isoform and is part of the inflammatory response, as its elimination in epithelial cells was reported to decrease proinflammatory gene expression [188]. P38α MAPK is also regarded as a “molecular switch for the activation of myogenic differentiation” [189]. In fact, p38α binds to—and regulates—many promoters during myogenesis [190]. While the factors that directly activate *DUX4* gene expression are still unknown [140], in vitro experiments demonstrated that clinically advanced p38α/β inhibitors are able to reduce *DUX4* expression without disrupting muscle differentiation [141]. Observations showed *DUX4* expression is exquisitely sensitive to p38 inhibition and requires much less inhibitor than in the case of blocking differentiation. According to Oliva et al. (2019), when xenograft mice had been treated with the p38 inhibitor losmapimod for 14 days, the total number of both human and mouse muscle cells increased compared to nontreated animals. The molecular mechanisms that tie *DUX4* expression to p38 activity remain to be elucidated [34]. However, due to these findings, the study “*ReDUX4*” was initiated by *Fulcrum Therapeutics*/ USA to assess “the safety and efficacy of losmapimod” in patients with FSHD1 [191]. Recruitment of volunteers for the trial started in August 2019 and enrollment was completed in February 2020. The trial involves 80 patients who are randomly allocated to either placebo or losmapimod [191]. Patients are given 15 mg of losmapimod or placebo twice a day as two 7.5 mg tablets per oral dose—for a daily total of 4 pills or 30 mg (clinicaltrials.gov: NCT04264442) [192].

It was envisioned that the primary endpoint of the study will be in the first quarter of 2021, and that all data—encompassing all secondary and exploratory endpoints—will be obtained in the second quarter of 2021. Results from the interim analysis in the first 29 randomized subjects indicate that *DUX4*-driven gene expression did not show a separation from placebo at 16 weeks. However, muscle biopsies with the highest levels of *DUX4* expression before the start of the trial showed a 38-fold reduction in *DUX4*-driven gene expression following treatment with losmapimod compared to a 5.4-fold reduction with placebo. According to *Fulcrum Therapeutics* the results indicate that muscle biopsies within the higher levels of *DUX4*-driven gene expression may be needed to observe a reduction from baseline [193].

#### miRNAs and AOs

Whilst vector-based approaches for RNA interference (RNAi) therapy with miRNAs “have remained largely in the pre-clinical realm”, antisense oligonucleotide (AO)-based approaches have seen more advancement [194]. AOs and miRNAs share a fundamental principle: they bind target RNA through Watson–Crick base pairing. Whilst AOs are developed as short synthetic single strands, miRNA functions as a duplex which is associated with a so-called RNA-induced silencing complex (RISC). In the latter case, one strand (the passenger strand) disappears and the remaining strand (the guide strand) interacts with RISC to bind complementary RNA. The differences between the two approaches show different strengths and weaknesses regarding drug development [195, 196].

#### miRNAs

The human organism uses miRNAs for gene silencing by creating small sequences with 19–25 nucleotides that target mRNA, thus inhibiting protein production [197]. miRNAs play an essential role in a large number of biological cellular processes such as development, cell differentiation, cell proliferation, or cell death [198]. Each miRNA can possibly regulate hundreds of different mRNAs, suggesting that a significant proportion of eukaryotic genes are regulated by miRNAs [199]. Wallace et al. (2018) recently tried to silence *DUX4* with adeno-associated viral vectors “to deliver targeted microRNA expression cassettes” (*miDUX4s*) [194]. They showed proof of concept for this procedure in mice, and made additional efforts to assess matters of safety associated with *miDUX4* overexpression and sequence-related off-target silencing. They reported improvements in vector design and enhancement of their *miDUX4* sequence repertory as well as differential toxicity—providing data to help advance RNAi gene therapy for FSHD [194].

The functionality of miRNAs has been researched extensively, but there remain several difficulties regarding "off-target effects" [200], toxicity [201], and undesirable immune responses [202]. The success of synthetically developed miRNAs highly depends on the availability of a safe and efficient transport system for the synthetically modulated RNA sequence into the cell [203].

#### AOs

While there have been promising studies regarding the use of AOs [204–206], there remains a need to more effectively knock down *DUX4* expression and screen for AOs against *DUX4.* Lim et al. (2020) used AO chemistries that directly degrade target mRNA and that do not passively act via mRNA processing interference. They worked with locked nucleic acid (LNA) gapmers to knock down *DUX4* pre-mRNA [207, 208]. LNA gapmers consist of a central segment of DNA flanked by short LNA stretches (see Fig. 8 (left)) They bind targets by sequence complementarity, producing a DNA/RNA hybrid that is cleaved by RNase H—which leads to gene knockdown [209]. LNAs are a class of modified RNA-nucleic acid analogous that carry a supplementary methylene bridge. This modification makes them resistant to nucleases and increases affinity to complementary RNA sequences [210]. Apart from sugar modifications, phosphorothioate backbones are used along the entire length of the AOs to provide further nuclease resistance [207, 211] when binding to RNA targets. Lim et al. (2020) targeted *DUX4* pre-mRNA [207, 208], which is generally processed through post-transcriptional modification such as capping, polyadenylation or splicing before it can leave the cell nucleus as mRNA and is available as a template for translation [212]. Lim et al. (2020) showed in vitro that LNA gapmer AOs successfully knock down *DUX4* in immortalized FSHD myoblasts and the *FLExDUX4* FSHD mouse model. They chose to mainly target *DUX4* exon 3, which is specifically associated with the pathogenic *DUX4* transcript (see “DUX4 gene expression”). They also found potential functional benefits of AOs on muscle fusion and structure in vitro. In vivo injection of one LNA reduced *DUX4* mRNA expression (by 84% one day after injection and 70% after repeating the experiment and collecting muscles seven days post injection). According to the authors, while the designed LNA gapmers could knock down *DUX4* expression in skeletal muscle significantly and selectively, effects on muscle structure and function, and an evaluation of the pharmacokinetic properties of LNA gapmers in vivo, remain to be determined with a systemic treatment study [207, 208].

#### CRISPR-Cas13

Another prospective treatment for FSHD regarding DNA level was introduced by Rashnonejad et al. (2019), who developed a new Cas13/CRISPR mediated *DUX4* mRNA silencing method which does not cleave DNA. The treatment can be accurately directed to a RNA transcript of interest utilizing a sequence-specific guide RNA (gRNA), thereby reducing the risk of permanent DNA damage. The authors targeted distinct parts of *DUX4* mRNA and showed their ability to markedly suppress *DUX4* and inhibit cell death in vitro and in vivo. According to the authors, “additional in vivo studies are underway” regarding the capabilities of the AAV-delivered CRISPR/Cas13 system [213].

#### G-quadruplex ligands

Ciszewski et al. (2020) have detected a number of G-quadruplexes (GQs) by using bioinformatic analysis of the genomic *DUX4* locus. These are shaping sequences and their presence was demonstrated “in synthetic oligonucleotiode sequences derived from the enhancer, promoter and transcript of *DUX4* through circular dichroism and nuclear magnetic resonance analysis.” [214]. The authors subsequently analysed the binding character of berberine, a naturally appearing GQ stabilizing compound, to these structures. An in vitro study in FSHD patient myoblasts was conducted using berberine as a treatment. Interestingly, there was a reduction of *DUX4* and its target genes. Additional analysis using a *DUX4*-mouse model validated the therapeutic impact of berberine on downregulating *DUX4* protein expression preventing muscle fibrosis, and hence rescuing muscle function [214].

#### Inhibition of HA biosynthesis

DeSimone et al. (2019) hoped to identify molecular pathways that mediate *DUX4* toxicity by using the *DUX4*-inducible human myoblast cell line *MB135-DUX4i*.

*MB135-DUX4i* has been demonstrated to have similar molecular disease pathologies such as FSHD myogenic cells. In contrast to FSHD cells, which sporadically express *DUX4* in only a few nuclei at different times (1 in every 200 to 1000 cells [179]), *MB135-DUX4i* cells can be simultaneously stimulated to induce high-level expression of *DUX4.* This enables molecular analysis of *DUX4* pathologies “under controlled and acute experimental conditions” [215].

The authors used proteomic coimmunoprecipitation (co-IP) assays and found that increased hyaluronic acid (HA) levels correlate with observed and several unobserved cellular pathologies. The latter include disturbance of the usually perinuclear localization of *C1QBP* (a HA-binding protein) and the mitochondria. It was demonstrated that *DUX4* expression supports accumulation of HA in *DUX4*-expressing cells and that multiple *DUX4*-induced molecular pathologies are mediated by HA which accumulates following *DUX4* induction. *DUX4*-expressing myoblasts, treated with the competitive HA biosynthesis-inhibitor 4-methylumbelliferone (4MU), inhibited *DUX4*-mediated accumulation of HA and the initiation of the pathologies without having an observable influence on *DUX4* protein amount or nuclear localization. These findings demonstrate HA as an important influencer of *DUX4* pathology that operates at an early stage in *DUX4* pathogenesis [215].

#### Inhibition of the cellular hypoxia response

A pathogenic connection to the cellular hypoxia response—which is the main influencer of *DUX4*-induced cell death (see “DUX4 downstream signalling: HIF1-α”)—was demonstrated by *c*onducting a genome-wide CRISPR-Cas9 screen to detect genes whose loss-of-function provide survival in case of *DUX4* expression*.* Studies that used the immortalized myoblast line *MB135-DUX4i*, combined with a doxycycline-inducible *DUX4* transgene to guarantee continuous *DUX4* expression in all cells, showed that the cellular hypoxia response can be disturbed with inhibitors of the phosphatidylinositol 3-kinase (PI3K)/Akt/mTOR [216] and Ras/mitogen-activated protein kinase (MAPK) signalling pathways [217] (see “Therapeutic approaches: p38 inhibition”-MAPK inhibitors are also used in context of β2AR signalling). When treating patient cells, PI3K/Akt/mTOR inhibitors showed more effectiveness at a lower dose and were thus selected for further analysis*.* Inhibition led to enhanced *DUX4* protein turnover and reduction of the cellular hypoxia response and apoptosis. Furthermore, FSHD disease biomarkers could be decreased in patient myogenic lines, whilst structural and functional attributes in two zebrafish models of FSHD could be improved [179].

### Current therapy approaches

#### Antioxidants

Antioxidant treatment of FSHD is considered a reasonable approach given that increased oxidative stress seems to be a central mechanism in this disease. Several reports demonstrate the beneficial effects of antioxidants on FSHD muscles in vitro, including a higher resistance against H_2_O_2_, reduced *DUX4*-induced toxicity and improved myotube formation [121, 218]. However, results are inconclusive in FSHD patients. Whilst a combinatory treatment with vitamin C, vitamin E, zinc, and selenium improved the maximum voluntary contraction and endurance of quadriceps in FSHD patients, results from a two-minute walk test, were insignificantly different [219]. Contrary to the above results, supplementation of methionine and folic acid (both potent antioxidants [220, 221] could not demonstrate a beneficial effect on muscle health or disease state [222]. Interestingly, a combined supplementation of docosahexaenoic acid, eicosapentaenoic acid, vitamin E, curcumin, acetyl-L-carnitine, vitamin C, coenzyme Q10, dry extract of the roots of scutellaria, and dry extract of green tea, demonstrated significant positive effects on a 6 min walk distance and the isokinetic knee extension in FSHD patients [223]. It should be noted that the sample size was less than 27 subjects per group in all studies, potentially masking any beneficial effect from the corresponding treatment. Therefore, conclusions from antioxidant treatments are unreliable given the low number of studies and small population sizes. Nevertheless, the limited beneficial effects observed provide a rationale to conduct larger multi-centred trials.

#### Aerobic exercise and strength training

Five cardiovascular training trials done by 111 FSHD participants proved the positive effects that aerobic exercise training has on patients with FSHD [224]. Muscle mass diminishes 3–8% per decade after the age of 30, and decreases at a higher rate after the age of 60. In this regard, exercise training and appropriate nutrition can have remarkable effects on muscle mass and strength [224–227]. Therefore, in the case of FSHD, exercising regularly is vital as untrained muscles accelerate the aging process and lead to reduced muscular resilience and chronic muscle pain [226]. Also, strength training—involving several repetitions with light weights [228]—is considered a valid option for FSHD patients. "High Intensity Interval Training “ (HIIT) should only be done under medical supervision [226]. Furthermore, training should be combined with physical therapy and adapted to individual needs in order to counteract physical overload [229]. A combination of aerobic exercise and strength training (and cognitive-behavioural therapy) should therefore be considered given the benefits that aerobic exercise has demonstrated [224, 230, 231]. In a study by Bankolé et al. (2016), sixteen FSHD patients were randomly split up in training (TG) and control (CG) groups (both n = 8) in a 6-month home-based training program. (The CG-patients also did an identical exercise intervention after this time period.) The training-schedule consisted of cycling 3 times a week for 35 min. Remarkable improvements could be shown in the peak oxygen uptake and maximal aerobic power by week 6—up to week 24. Improvements in muscle endurance, maximal quadriceps strength, and 6-min walking distance was demonstrated. Moreover, fatigue was reported to decrease. “Muscle fibre cross-sectional area and citrate synthase activity increased by 34% and 46%, respectively.” [231]. However, according to Voet et al. (2019), many studies remain insufficient for subgroup analyses (regarding patients with severe or mild phenotype), sample size, duration, or evaluation of dose–response relationships. Additional research with strong methodology and more participants is required [224].

## Conclusion

The aim of this review is to provide an overview of FSHD regarding genetics, pathophysiology, currently discussed therapy approaches, and future aspects of methodology. There remains potential in the role of modifier genes that have not been identified yet, looking at direct D4Z4 binding proteins [140] or gender specific effects of *DUX4* signalling [133]. An early intervention of DNA levels in order to minimize *DUX4-*effects may have the most positive results given that *DUX4* is believed to induce hundreds of different target genes [28, 119]. In this context, the locus of the 4q telomere at the nuclear envelope [117] and the long distance interactions of D4Z4 seem to be particularly important in disease progression considering both methylation levels of the 4q35 locus and a changed gene expression [60, 118] beyond *DUX4* expression.

Also, further research of signalling pathways behind the effect of β2 adrenergic receptor-agonists showed that p38 mitogen-activated protein kinase is a mediator of *DUX4* expression [34]. Use of LNA gapmer AOs recently lead to the successful inactivation of *DUX4* at pre-RNA levels [207, 208].

Investigations into the CRISPR/Cas13 system are currently being conducted [213].

It was found that *DUX4* protein uses different systems such as the hyaluronic acid pathway [215] and G-quadruplexes [214] for further action. Although chemical inhibitors have been shown to suppress *DUX4* effects, further research is needed to build upon the new findings. Researchers are beginning to use screens of small molecules to discover drugs that influence D4Z4 methylation or translation of *DUX4*. Regarding *DUX4* targets, ROS seem to have an important role in alleviating the effects of *DUX4* toxicity in the disease mechanism, whilst current research has linked the main driver of apoptosis to cellular hypoxia response [179]. The growing pace of drug development has generated an urgent requirement for clinical trial readiness [108]. The relevance of clinical trial planning is prominent, and standardization is becoming more necessary. Current therapy approaches try to counteract muscle loss and weakness. In this context, antioxidants combined with aerobic exercise and light strength training are considered to be vital in alleviating *DUX4* toxicity [223, 226, 231]. However, in this regard, several studies use small sample sizes whilst specifications regarding the examination criteria remain unclear [224]. Therefore, the international multicentre study ReSolved (March 2018—March 2022) may result in more efficient clinical trial designs [108]. Against this background, further research should focus on disease severity (in relation to age and gender), pre-exercised muscle, lifestyle, affected muscle groups regarding pain, quality of life, and the individual genetics. FSHD is a genetically complex type of muscular dystrophy. Therefore, detailed information about study participants, categories regarding disease severity, and standardization of methodology may significantly help to interpret results.

## Outlook

Patient registries, biomarkers (see “Disease monitoring”), and clinical outcome measures (see “Trial readiness”) have to be included in order to facilitate targeted therapy and diagnostics. Disease progression can be adequately monitored by using standardized clinical evaluation such as the Comprehensive Clinical Evaluation Form (CCEF) [232–234] in combination with parameters like the clinical severity score by Ricci et al. (“Ricci score”) [233] and the FSHD clinical score [235]. These tools are especially important for the standardization of follow-up-studies. Also, family studies must be conducted in order to elucidate the extent of disease variability.

It is uncertain if methylation analysis is a useful tool for FSHD diagnosis and prognosis given that the factors which influence the methylation status of the D4Z4 macrosatellites are currently not fully understood. In this regard, Nikolic et al. (2020) observed a high variable distribution of D4Z4 methylation whilst investigating D4Z4 methylation status at 4q35 in a large cohort of patients through *methylation-sensitive restriction enzymes 1* (*MRSE1*)[58]. Therefore, methylation techniques per se are not sufficient. Exactly how the nuclear lamina impacts on global chromatin architecture needs to be further examined. In FSHD, the short D4Z4 array seems to interrupt interaction amonst D4Z4, the telomere, and the nuclear periphery (which is usually organized via long distance loops, encompassing 2 ToADs in the context of a higher order chromatin organisation). The altered gene expression in this regard involves genes such as *FAT1* and *SORBS2,* which should be included in further investigations as both genes have been shown to play a role in skeletal muscle [60, 116, 118].

Stakeholders should prioritize collaboration and commitment of resources in order to develop an all-encompassing FSHD model that includes most muscle phenotypes in a single animal [4]. However, attempts to model FSHD in animal models are difficult since the pathophysiological mechanisms of *DUX4* expression are not fully understood. No model has yet been able to encompass all the characteristic effects of *DUX4* expression regarding differently affected muscle parts, extramuscular manifestations, right-left asymmetry, or gender-specific effects. Therefore, it might be useful to analyse and use comparable parameters within different organisms (see “Animal models”)*.* Neverthlesss, differences in animal models are currently inevitable especially given that FSHD is a disease for which non-primate animal models remain imperfect.

Recently a Cas13/CRISPR mediated *DUX4* mRNA silencing method has been developed which targets RNA (not DNA) and can be specifically directed to a RNA transcript of interest utilizing a sequence-specific guide RNA [213]. This, in combination with the strategies illustrated in this review, seems to be a promising approach especially as it makes Cas13 a potentially significant therapy for influencing gene expression without altering genome sequence. Additional in vivo studies are underway regarding the capabilities of the AAV-delivered CRISPR/Cas13 system. The results of this method may be an exciting field for future investigations.

## Data Availability

Data sharing is not applicable to this article as no datasets were generated or analysed during the current study.

## Abbreviations

3D FISH: Three-dimensional fluorescent in situ hybridization
4MU: 4-Methylumbelliferone
3′UTR: Three prime untranslated region
β2-AR: β2-Adrenergic receptor
ACSL1: Acyl-CoA synthetase long chain family member 1
AO: Antisense oligonucleotide
AFO: Ankle-foot orthotic
ARNT: Aryl hydrocarbon receptor nuclear translocator
ATPase: Adenyltriphosphatase
BAMS: Bosma arhinia microphthalmia syndrome
Bmp2: Bone morphogenetic protein 2
cAMP: Cyclic adenosine monophosphate
CBP: CREB-binding protein
CCEF: Comprehensive clinical evaluation form
CDKN1A: Cyclin dependent kinase inhibitor 1A
CG: Control group
ChIP-seq: Chromatin immunoprecipitation DNA-sequencing
CIS: Checklist individual strength
CO_2_: Carbon dioxide
COA: Clinical outcome assessment
Co-IP: Coimmunoprecipitation
CRISPR: Clustered regularly interspaced short palindromic repeats
CRYM: Crystallin μ
CTCF: CCCTC-binding factor
DNMT3B: De novo methyltransferase 3B
dsRNA: Double-stranded RNA
DUX4: Double Homeobox 4
DUX4-FL: DUX4-full-lengths
DUX4-S: DUX4-short
EcoRI: Restriction endonuclease I from *Escherichia coli*
EIF2AK2: Eukaryotic translation initiation factor 2 alpha kinase 2
EIM: Electrical impedance myography
FAT1: FAT atypical cadherin 1
FSHD: Facioscapulohumeral muscular dystrophy
FSHD-COM: FSHD composite outcome measure
GPX4: Phospholipid hydroperoxide glutathione peroxidase
GQ: G-quadruplex
GSH: Glutathion
GSSG: Glutathione disulfide
GSTA4: Glutathione S-transferase A4
GSTO1: Glutathione S-transferase omega-1
H_2_O_2_: Hydrogen peroxide
H3K9me3: Tri-methylation at the 9th lysine residue of the histone H3 protein
HA: Hyaluronic acid
HIF1A: Hypoxia-inducible factor 1-alpha
HIIT: High intensity interval training
HP1: Heterochromatin protein 1
ICF-syndrome: Immunodeficiency, centromeric instability, and facial anomalies syndrome
IL: Interleukin
KAFO: Knee-ankle-foot orthotic
KMTase: Lysine methyltransferase
LAD: Lamin associated domain
LNA: Locked nucleic acid
MAPK: Mitogen activated protein kinase
MB135-DUX4i: DUX4-inducible human myoblast cell line 135
miDUX4s: MicroRNA expression cassettes
miRNA: MicroRNA
MMT: Manual muscle testing
MRF: Myogenic regulatory factors
MRF4: Muscle regulatory factor 4
MRI: Magnetic resonance imaging
mRNA: Messenger RNA
MRSE1: Methylation-sensitive restriction enzymes 1
mTOR: Mammalian target of rapamycin
MuRF1: Muscle RING-finger protein-1
Myf5: Myogenic factor 5
Myf6: Myogenic factor 6
MyoD: Myogenic differentiation 1
MyoG: Myogenin
NADPH: Nicotinamide adenine dinucleotide phosphate
NIV: Non-invasive ventilation
NLS: Nuclear localization signal
NMD: Nonsense-mediated mRNA decay
ORF: Open reading frame
PI3K: Phosphatidylinositol 3-kinase
PAS: Polyadenylation signal
PAX3: Paired Box 3
PAX7: Paired Box 7
PITX1: Paired-like homeodomain transcription factor 1
PKA: Protein kinase A
PPTase: Phosphatase
QMT: Quantitative myometry
Ras: Rat sarcoma
RISC: RNA-induced silencing complex
RNAi: RNA interference
RNase H: Ribonuclease H
RNASEL: Ribonuclease L
ROS: Reactive oxygen species
RT-PCR: Reverse transcription polymerase chain reaction
SMCHD1: Structural maintenance of chromosomes flexible hinge domain containing 1
SNP: Single nucleotide polymorphism
SOD: Superoxide dismutase
SORBS2: Sorbin and SH3 domain-containing protein 2
Sp1: Specificity protein 1
STIR: Signal on short-tau inversion recovery
ToAD: Topologically associating domain
TAD: Transcription-activating domain
TG: Training group
TIC-DUX4 model: Tamoxifen-inducible-DUX4 model
TNF-α: Tumor necrosis factor alpha
UPF1: Up-frameshift protein 1
VNTR: Variable number tandem repeat
WWC2: WW and C2 domain containing 2
